# Toward precision oncology in LUAD: a prognostic model using single-cell sequencing and WGCNA based on a disulfidptosis relative gene signature

**DOI:** 10.3389/fimmu.2025.1581915

**Published:** 2025-05-21

**Authors:** Panpan Li, Han Zhang, Limin Sun, Xiaojuan Wu

**Affiliations:** ^1^ Department of Pathology, School of Basic Medical Sciences and QiLu Hospital, Shandong University, Jinan, Shandong, China; ^2^ Tianjin Chest Hospital, Tianjin University, Tianjin, China; ^3^ Department of orthopedics, Shandong provincial third hospital, Jinan, Shandong, China

**Keywords:** disulfidptosis1, LUAD2, TME3, immunotherapy4, ScRNA-seq5

## Abstract

**Background:**

Disulfidptosis, a recently identified mechanism of cell death characterized by intracellular sulfide accumulation, leading to cellular exhaustion. Our objective is to create a prognostic model using a cohort of disulfidptosis-related genes (DRGs) to assess their prognostic value in lung adenocarcinoma (LUAD). This research not only deepens our understanding of the molecular mechanisms underpinning LUAD but also offers promising avenues for new clinical treatment biomarkers and therapeutic targets.

**Methods:**

We employed various methodologies to assess DRGs in LUAD. Gene expression in single cell RNA sequencing (scRNA-seq) data was assessed using the AUcell algorithm. In the TCGA [LUAD] dataset, disulfidptosis-related enrichment scores were calculated using ssGSEA, and core gene sets were identified through the Weighted Gene Co-expression Network Analysis (WGCNA) algorithm. Differential gene analysis was conducted using the limma package and intersected with core gene sets. Univariate Cox regression analysis revealed genes with significant effects on LUAD prognosis. A prognostic model was developed using LASSO and Cox regression, utilizing median model scores for stratifying patient risk. Kaplan-Meier curves assessed prognostic differences between risk groups. Comprehensive analyses were performed on the tumor microenvironment (TME) and mutational landscape across different risk groups. Immune response characteristics and functional enrichment patterns were further evaluated in these cohorts.

**Results:**

Our study delved into disulfidptosis in LUAD through a series of analyses: scRNA-seq data processing, WGCNA analysis, construction of a prognostic model, evaluation of clinical features and risk, enrichment analysis, mutation landscape assessment, and examination of the tumor microenvironment. We identified core genes related to disulfidptosis and established a prognostic model to classify patients based on risk scores. Notable differences in TME characteristics, immune cell infiltration, mutation landscape, and biological pathway activities were observed between risk groups, shedding new light on LUAD clinical treatment and biomarker discovery. Cell experiments highlighted the significance of KCNK1 in LUAD cells, suggesting its potential as a therapeutic target.

**Conclusion:**

A prognostic model centered on DRGs was effectively developed to predict prognosis of LUAD and immunotherapy response. Our initial investigations unveiled KCNK1’s oncogenic role in LUAD, identifying it as a potential therapeutic target.

## 1 Introduction

With advancements in modern medical technologies, the incidence of lung cancer in males has demonstrated an annual decline of 2.5% since 2006, while the female incidence rate has decreased by 1% per annum. Nevertheless, lung cancer remains a predominant global health concern, with LUAD being the most prevalent subtype (1, 2). In 2024, an estimated 234,580 new lung cancer cases are projected (116,310 males; 118,270 females), accompanied by approximately 125,070 deaths (65,790 males; 59,280 females). The 3-year survival rate for small cell lung cancer (SCLC) has shown only marginal improvement from 9% to 13%, whereas the relative 3-year survival rate for non-small cell lung cancer (NSCLC) increased from 26% in 2004 to 40% in 2017. Recent studies underscore its significant impact on public health, shaped by factors like smoking, environmental exposures, and genetic predispositions (3). Smoking-associated lung cancer accounts for 81% (101,300 cases) of projected lung cancer mortality in 2024, while non-smoking-related etiologies contribute approximately 20,300 fatalities, encompassing environmentally induced subtypes and genetically driven variants such as EGFR-mutant adenocarcinomas (4). The latest epidemiological insights from 2023 highlight lung cancer as a leading cause of cancer incidence and mortality worldwide (5). Despite advancements in treatment and early detection, the survival rates for lung cancer, especially for LUAD, continue to be relatively low (6). The variations in incidence, mortality, and survival rates of lung cancer across diverse regions and populations underscore the complex interactions of socioeconomic, racial, and environmental determinants. This evolving landscape underscores the urgency for targeted interventions and continued research in understanding and combating this disease.

Programmed cell death (PCD) represents a crucial physiological process, playing a role in maintaining the homeostasis of the internal tissue environment and eliminating damaged or unnecessary cells in the organism. PCD encompasses multiple forms, such as apoptosis, autophagy, cuproptosis, ferroptosis, disulfidptosis, etc (7).Notably, the majority of cancer treatments exert their anti-tumor effects by activating apoptosis (8). Ferroptosis is a natural anti-tumor mechanism that exhibits its tumor-suppressive function through interactions with various tumor suppressor genes (9). Cuproptosis genes are correlated with the infiltration levels of multiple immune cells and the sensitivity of cancer cells to various drugs (10).

Disulfidptosis is a novel form of cell death characterized by the abnormal formation of protein disulfide bonds within cells and typically arises under conditions of heightened oxidative stress (11–13). Studies have demonstrated that disulfidptosis is mediated through the actin cytoskeleton’s responsiveness to disulfide bond stress (13). This stress triggers excessive disulfide bond formation among cysteine residues, subsequently altering protein structure and functionality. Central to disulfidptosis is the disruption of intracellular signaling and metabolic pathways, ultimately leading to cell dysfunction and demise. This process underscores the intricate relationship between oxidative stress, protein homeostasis, and cellular mortality. In the realm of cancer therapy, metabolic treatments employing GLUT inhibitors have shown promise. These inhibitors can induce disulfidptosis and have been observed to hinder tumor growth, representing a novel approach in cancer management (14). Although prognostic models based on disulfidptosis have been developed for bladder cancer, its implications and effectiveness in LUAD are still relatively uncharted, pointing to an area ripe for further research.

In this study, our initial goal is to identify a collection of DRGs within the context of LUAD. Our objective is to construct a prognostic model utilizing these genes to predict outcomes in LUAD and assess the efficacy of immunotherapy. By stratifying patients according to this risk model, we methodically examined variations in prognosis, TME, mutation profiles, and responses to immunotherapy across different risk categories. Additionally, we fused clinical characteristics with risk scores to forge an innovative, comprehensive prognostic tool specifically for LUAD. Through this research, we aspire to enrich our understanding of disulfidptosis in LUAD and to open up novel avenues for its treatment.

## 2 Methods

### 2.1 Data sources

ScRNA-seq data from 10 primary LUAD patients were acquired from Code Ocean(https://codeocean.com/capsule/8321305/tree/v1). For the training set, gene expression, clinical, and mutation information of TCGA [LUAD] patients were sourced from The Cancer Genome Atlas (TCGA; https://portal.gdc.cancer.gov/). We also downloaded four independent GEO cohorts for the training set from the Gene Expression Omnibus (GEO; http://www.ncbi.nlm.nih.gov/geo/). The bladder cancer immunotherapy cohort data were obtained from http://research-pub.gene.com/IMvigor210CoreBiologies/.

### 2.2 ScRNA-seq data processing

ScRNA-seq data processing utilized the ‘Seurat’ R package (15–18). We first filtered out low-quality cells, keeping those with nFeature ranging from 500-10000, nCount from 1000-100000, mitochondrial gene percentage (pMT) of 0-30%, and hemoglobin gene percentage (pHB) of 0-5%. The CellCycleScoring function assessed cell cycle impacts, and SCTransform was used for data scaling and normalization. RunPCA performed principal component analysis. Using the t-SNE algorithm (19), we identified significant clusters with the first 20 principal components, and 25 cell clusters were classified using the FindNeighbors and FindClusters functions. Cell annotation was performed using SingleR and manual methods. After reviewing literature, 16 DRGs were identified, and their scRNA-seq activity was evaluated with the ‘AUCell’ package (11). Cells were categorized into high and low activity based on median scores, and differential genes were identified using FindAllMarkers. Based on the CellChat R package, the intercellular communication patterns between high and low disulfidptosis activity groups were evaluated.

### 2.3 Gene co-expression network analysis

The ssGSEA algorithm was applied to compute enrichment scores for each TCGA [LUAD] patient using differential genes between high and low disulfidptosis activity groups (20, 21). The WGCNA algorithm computes a weighted gene co-expression network (22). We standardized gene expression data from TCGA [LUAD] tumor samples to ensure consistency. Quality control utilized the goodSamplesGenes function from the WGCNA package to eliminate samples and genes with significant missing data or high expression variability. We then selected an optimal soft threshold through the pickSoftThreshold function, crucial for constructing a robust co-expression network. This step involved calculating the scale-free topology fit index across various thresholds, aiming to enhance the network’s scale-free properties while balancing average connectivity. Gene expression data were transformed into an adjacency matrix and subsequently into a Topological Overlap Matrix (TOM) to evaluate gene co-expression strengths. Using hierarchical clustering and dynamic tree cutting methods (cutreeDynamic), we identified co-expressed gene modules, indicative of highly correlated genes within the network and their potential biological functions. For each module, we calculated the module eigengene (ME) and assessed gene significance (GS), determining the association of each gene with external traits, including disease status or clinical phenotype. Pearson correlation analysis was used to evaluate the associations between module eigengenes and clinical traits, identifying gene modules significantly linked to key clinical features. Lastly, we visualized these gene modules and conducted additional bioinformatics analyses to explore their associations further.

### 2.4 Development of the prognostic model

Initially, differential gene expression between TCGA [LUAD] tumors and normal samples was identified using criteria: p < 0.05, logFc > 1. The identified differential genes were then cross-referenced with disulfidptosis-associated gene modules, ascertained through WGCNA calculations. Univariate Cox regression analysis was conducted on the intersected genes to identify those with significant prognostic relevance for TCGA[LUAD] patients. Next, LASSO regression((λ=0.0258) was utilized to further refine genes related to prognosis, culminating in the development of the final prognostic model via integrated multivariate Cox regression. Kaplan-Meier curves were used to assess the impact of risk scores on prognosis, while the ‘TimeRoc’ package evaluated their diagnostic capability.

### 2.5 Development of the nomogram

Primarily, an assessment was conducted on the distribution of various clinical features across different risk groups. Following the initial analyses, univariate and multivariate Cox regression analyses were performed on selected clinical features and risk scores to identify significant variables for nomogram development. The construction of the nomogram was carried out using the ‘rms’ R package (23). The effectiveness of the nomograms was validated using receiver operating characteristic (ROC) curves, calibration curves, and Decision Curve Analysis (DCA) (24, 25).

### 2.6 Enrichment analysis

Gene Set Enrichment Analysis (GSEA) was conducted utilizing TCGA [LUAD] expression profile data and risk score files (26, 27). Data were firstly imported from corresponding text files, undergoing essential preprocessing that included data cleaning and formatting. Subsequently, data averaging was performed utilizing the avereps function within the limma package. Following this, samples were categorized into high-risk and low-risk groups according to their respective risk scores. Average gene expression in both risk groups was computed and log-transformed, yielding the log fold change (logFC). These logFC values facilitated GSEA in identifying disease-associated biological pathways via functions available in the clusterProfiler package. Significantly enriched gene sets, selected based on p-values, were visualized utilizing the gseaplot2 function from the enrichplot package. Subsequently, the GSVA algorithm was employed to calculate significantly enriched pathway information in the high-risk group relative to the low-risk group (28). Finally, the ssGSEA algorithm was utilized to evaluate variations in immune cell proportions and immune function between high and low-risk groups.

### 2.7 Mutation analysis

Mutation data for TCGA [LUAD] were obtained using the ‘TCGAbiolinks’ R package and processed with ‘maftools’ after generating mutation annotation format (MAF) files (29, 30). The oncoplot function visualized mutation landscapes for high- and low-risk groups, and the somaticInteractions function clarified gene co-mutation relationships. Survival disparities among different risk groups and between high and low tumor mutation burden (TMB) levels were evaluated using the ‘survival’ and ‘survminer’ packages (31).

### 2.8 Tumor microenvironment assessment

This study utilized the ‘ESTIMATE’ R package to assess tumor sample purity and immune cell composition within the cellular matrix, resulting in the ESTIMATE Score, Immune Score, and Stromal Score (32). Subsequently, we obtained immune infiltration data from the Timer2.0 database (http://timer.cistrome.org/) and employed the ‘pheatmap’ R package to depict the differences in immune cell infiltration between high-risk and low-risk groups (33). This analytical approach facilitated our exploration of immune cell distribution patterns within TME across different risk strata.

### 2.9 Evaluation and validation of immunotherapy efficacy

This study requires analyzing the differences in immune checkpoint and MHC related gene expression between high-risk and low-risk tumor samples in the TCGA[LUAD] database, employing the Wilcoxon rank-sum test to ascertain the potential influence of these gene expression traits on patient stratification. To investigate the interplay between gene expression levels, risk scoring, and Hub genes, we computed the Spearman rank correlation coefficient. The outcomes of these analyses were visualized through the ggplot2 package in R, enhancing the clarity and intuitiveness of the assessment of variances and correlations. Furthermore, we downloaded Immunophenoscores (IPS) from the Cancer Immunome Atlas (TCIA) (https://tcia.at/home) database, comparing IPS scores among various risk groups via t-tests to gain insights into patient responsiveness to immune checkpoint inhibitor therapies (34). Ultimately, we confirmed the prognostic capability of our model and the variance in risk scoring among different immunotherapy responses (CR is an abbreviation for Complete Response, PR denotes Partial Response, PD signifies Progressive Disease, and SD represents Stable Disease) in two distinct immunotherapy cohorts, IMvigor210 and GSE7822.

### 2.10 Cell lines and culture

The human LUAD cell lines A549, PC9, and NCI-H1299, along with the normal bronchial epithelial cell line BEAS-2B, were procured from the American Type Culture Collection (ATCC). These cells were routinely cultured in RPMI-1640 medium, supplemented with 10% fetal bovine serum (FBS), and maintained in a controlled environment featuring a humidified atmosphere of 5% CO_2_ at 37°C. The identity of all cell lines was verified through short tandem repeat (STR) analysis and cross-referenced with the ATCC and Cellosaurus databases. The identification was carried out by Zhou Qiao Xin zhou Biotechnology Co., Ltd (Shanghai, China). Additionally, all cell lines were assessed for mycoplasma contamination using the MycAwayTM Plus-Color One-Step Mycoplasma Detection Kit, with results indicating no presence of mycoplasma.

### 2.11 Small-interfering RNA transfection

For siRNA transfection, we utilized sequences specified in **Supplementary Table 1**, procured from GenePharma (Shanghai, China). Cells were plated at a density of 2×10^5^ cells per well in six-well culture plates. Subsequent to seeding, cells were transfected with siRNA using Lipofectamine 2000 (Invitrogen, Carlsbad, CA, USA) following the manufacturer’s instructions.

### 2.12 RNA isolation and quantitative PCR analysis

RNA was isolated from the cultured cells utilizing the RNA-Quick Purification Kit (Yishan, Shanghai, China), ensuring integrity and purity for subsequent analyses. The conversion of extracted RNA to cDNA was achieved through the First Strand cDNA Synthesis Kit (Toyobo, Osaka, Japan), preparing the samples for amplification. RT-qPCR was performed using the ABI Prism 7000 Sequence Detection System. This process involved the use of SYBR Premix Ex Taq (Takara, Otsu, Japan) for the detection and quantification of gene expression. The primer sequences employed for the target genes are detailed in **Supplementary Table 1**. GAPDH was utilized as a reference gene to normalize the expression data and ensure accurate comparative analysis.

### 2.13 Western blot analysis

Cells were collected, pelleted, and lysed with CelLyticTM MT Cell Lysis Reagent (Sigma, St. Louis, USA) to extract total protein, which was then quantified using a BCA Reagent Kit (Beyotime, Shanghai, China). The primary antibodies of anti-KCNK1(Abways, AY2763; 1:800 dilution) and anti-GAPDH (HUABIO, EM1901-57; 1:1000 dilution) were used, with the latter serving as the loading control. Blots were then incubated with HRP-conjugated goat anti-rabbit/mouse IgG H&L secondary antibodies (Abways, AB0101/AB0102; 1:5000 dilution). The detection of protein bands was achieved using an Enhanced Chemiluminescence Kit (Millipore, Darmstadt, Germany).

### 2.14 Cell proliferation assay

The assessment of cell proliferation was conducted utilizing two distinct approaches: the Cell-Light™ EdU DNA Cell Proliferation kit (Ribobio, Guangzhou, China) and the Cell-Counting Kit-8 (CCK-8) (Targetmol, Shanghai, China). For the EdU incorporation assay, cells were plated at a density of 10,000 cells per well in 96-well plates. Post-EdU incorporation, staining was performed using 100 µL of the Apollo reaction mixture and 100 µL Hoechst 33342 for nuclear staining. Fluorescence microscopy (Olympus, Japan) was employed to visualize the stained cells. Quantify EdU stained positive cells to estimate cell proliferation rate. Concurrently, the CCK-8 assay was implemented as an alternative measure of cellular proliferation. A density of 3000 cells per well was established by seeding cells in a 96-well plate. The assay procedure followed the guidelines provided by the manufacturer. This method allowed for an additional quantitative evaluation of cell proliferation.

### 2.15 Assessment of colony formation capability

In the colony formation assay, a count of 2,000 cells was seeded into each well of a 6-well plate, followed by an incubation period of 15 days. After this duration, the cells were fixed using 4% paraformaldehyde for 15 minutes. Subsequent to fixation, staining was carried out using Crystal violet (Solarbio, China) to visualize the colonies.

### 2.16 Cell migration and invasion assays

To evaluate the migration and invasion ability of LUAD cells, Transwell assay was used, using inserts with a pore size of 8.0 µ m in a 24 well plate format (Corning, NY, U.S.). For invasion assessment specifically, the Transwell inserts were pre coated with Matrigel (BD Science, Sparks, MD, U.S.). A total of 25,000 LUAD cells, suspended in a serum-free medium, were introduced into the upper chambers, whereas the lower chambers were supplemented with a complete medium to serve as a chemoattractant. After a 24-hour incubation, cells that migrated or invaded through the pores were fixed with 4% paraformaldehyde and stained using Crystal violet (Solarbio, China) for visualization and counting.

### 2.17 Apoptosis analysis via flow cytometry

Apoptosis in A549 and PC9 cells was assessed using flow cytometry following a specific treatment regime. Post 48-hour transfection, cells were exposed to 2 µL of 10 µM cisplatin for therapeutic intervention, while the control group was treated with 2 µL of DMSO for 4 hours. Subsequently, the cells were collected utilizing a pancreatic enzyme solution devoid of EDTA and were resuspended in binding buffer at a concentration of 1×10^6^ cells per mL. The cells were then stained using FITC-Annexin V and propidium iodide (PI), utilizing the FITC Annexin V Apoptosis Detection Kit I (BestBio, Shanghai, China). This staining facilitated the quantification of apoptotic cells via flow cytometry, adhering to the instructions provided by the reagent kit. Reproducibility was ensured by conducting all experimental runs in triplicate.

### 2.18 ROS detection

ROS generation was assessed using a ROS Assay Kit (Beyotime, China). 48 hours after transfection, cells were stained with 10 µM DCFH-DA at 37°C for 30 minutes and subsequently analyzed using a flow cytometer.

### 2.19 Statistical analysis

Statistical analysis for the bioinformatics part was conducted in R, while the basic experimental part was analyzed using Graphpad and Image J. Differences between groups were analyzed using one-way or two-way ANOVA (Analysis of Variance). Survival analysis was performed using the Kaplan-Meier method, and differences in survival between groups were tested using the Logrank test. Correlations were assessed with the Pearson coefficient, considering p-values below 0.05 as statistically significant.

## 3 Results

### 3.1 Processing of scRNA-seq

**Figure 1:**
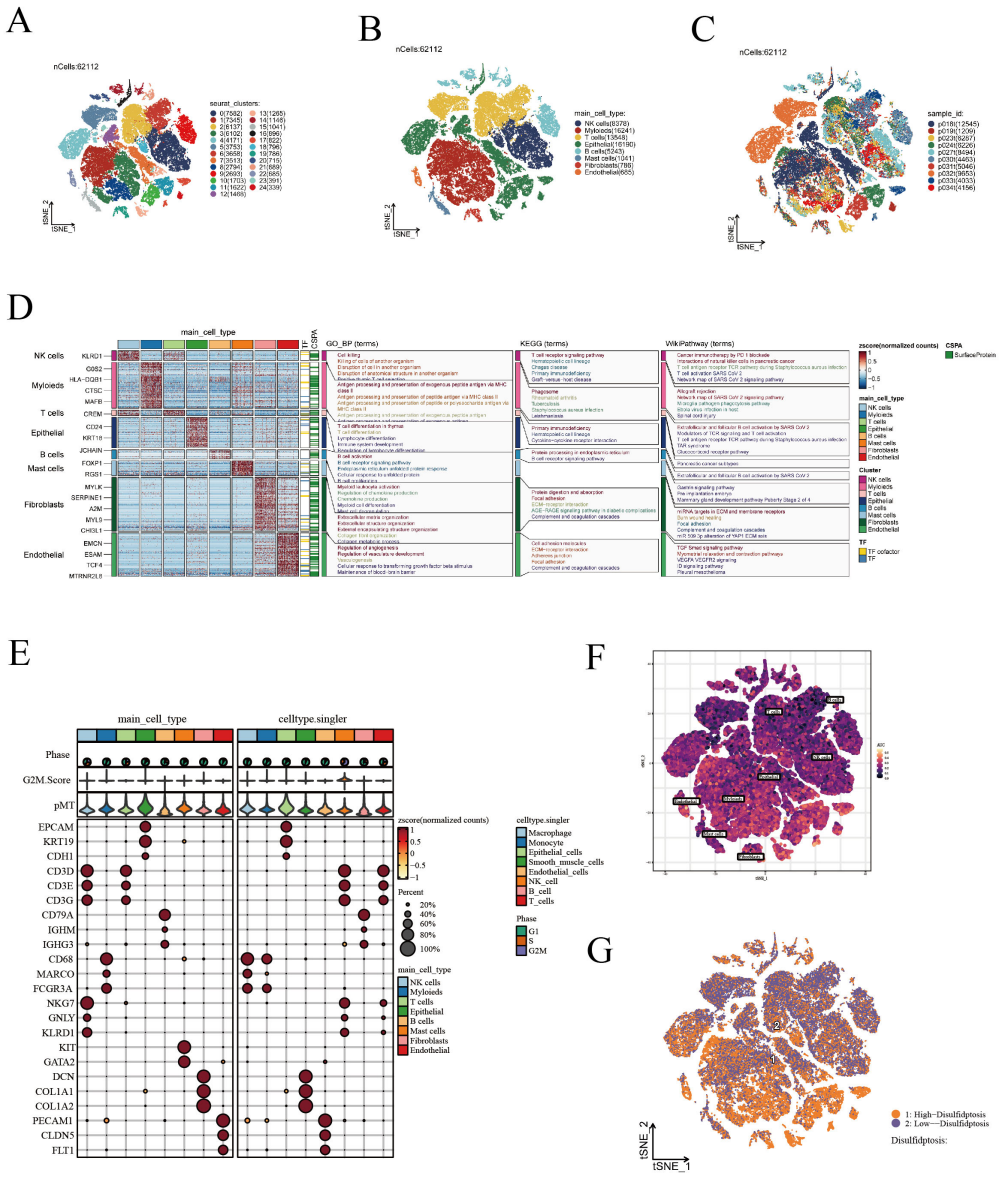
Visualization of scRNA-seq data. **(A)** t-SNE analysis identifying 25 unique cell subgroups. **(B)** t-SNE annotated visualization depicting 8 cell subgroups. **(C)** t-SNE plots demonstrating the distribution of cells from various sources. **(D)** Gene expression patterns and enrichment analysis across different cell types. **(E)** Display of marker genes. **(F)** Evaluation of DRGs activity in scRNA-seq using the AUcell algorithm. **(G)** t-SNE representation of cells with high and low DRS activity.

In this study, we isolated 15,575 high-quality cells after applying stringent quality control measures, as described in the methodology section and shown in **Supplementary Figure 1**. As demonstrated in **Figure 1A**, we conducted dimensionality reduction and clustering on all cells, categorizing them into 25 distinct cell subgroups. Subsequently, using a blend of manual annotation and the SingleR package, we classified the cells into 8 specific cell subgroups (**Figure 1B**). **Figure 1C** illustrates the sample origin of each cell, revealing a uniform distribution without noticeable batch effects. Thereafter, we assessed gene expression patterns and performed enrichment analysis for various cell types, including the high expression of KLRD1 in NK cells linked to cell killing and the T cell receptor signaling pathway (**Figure 1D**). **Figure 1E** and **Supplementary Figure 1E** display the expression of marker genes, further substantiating the precision of our cell annotation. Following an extensive literature review, we identified 16 disulfidptosis-related genes. Utilizing these genes, we applied the AUCell algorithm to evaluate cellular activity in single-cell data (**Figure 1F**). Cells were stratified into high and low disulfidptosis activity groups according to the median values of cellular activity scores (**Figure 1G**), followed by employing the FinAllmarkers function to identify differential genes between these groups. Our analysis of intercellular communication revealed that cells with heightened disulfidptosis activity participate in more frequent and intense cellular interactions, as evidenced by the increased number and strength of pathways (**Supplementary Figure 2A**). In the low disulfidptosis group, the SPP1 and MHC-I pathways were more active, while the EGF and Periostin pathways showed increased activity in the high disulfidptosis group (**Supplementary Figure 2B**). Moreover, among cells with high disulfidptosis activity, fibroblasts displayed the most intense communication, while epithelial cells exhibited the greatest number of communication pathways (**Supplementary Figure 2C**).

### 3.2 Gene co-expression network analysis

**Figure 2:**
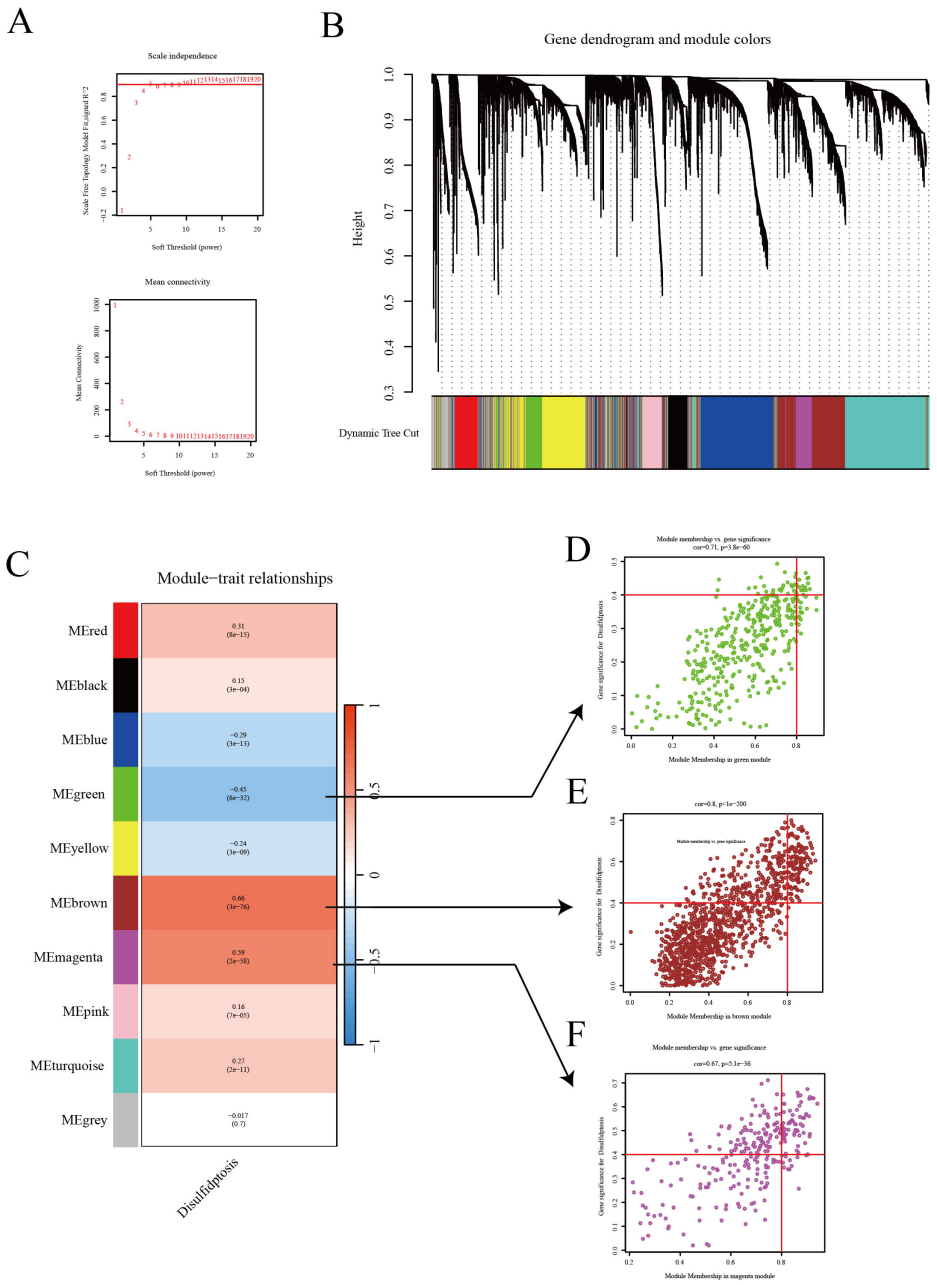
WGCNA reveals the relationship between gene expression patterns and Disulfidptosis. **(A)** Network topology analysis to establish an appropriate soft threshold, constructing a scale-free network reflecting the intrinsic structure of gene expression data. **(B)** Dendrogram and module colors illustrating the natural clustering of gene expression data. **(C)** Module-trait relationships showcasing the correlation of each module with disulfidptosis traits. **(D–F)** Scatter plots for three modules (green, red, purple) displaying the correlation of genes with module eigengenes.

In this study, we employed WGCNA to successfully identify gene modules that are closely associated with the characteristics of disulfidptosis in LUAD. To establish a scale-free network, a soft threshold of β=5 was applied (**Figure 2A**), ensuring the network topology conformed to a scale-free distribution. Following this, a gene dendrogram (**Figure 2B**) was employed to cluster genes according to similarities in their expression patterns, with distinct modules being differentiated by various colors. Further analysis of module-trait relationships (**Figure 2C**) uncovered correlations between individual modules and disulfidptosis characteristics, with the green module (MEgreen) exhibiting the strongest negative correlation, while the blue (MEblue) and brown (MEbrown) modules demonstrated the most pronounced positive correlations. Expounding upon these findings, we detailed the correlations between genes in the aforementioned three modules and disulfide death characteristics using scatter plots (**Figures 2D–F**). In these plots, each point represented a gene, with its position indicating its significance in the network and its association with disulfidptosis characteristics. These insights underscore potential biomarkers, offering invaluable resources for advancing research into the biological attributes of LUAD and enhancing immunotherapy strategies.

### 3.3 Construction of prognostic model

**Figure 3:**
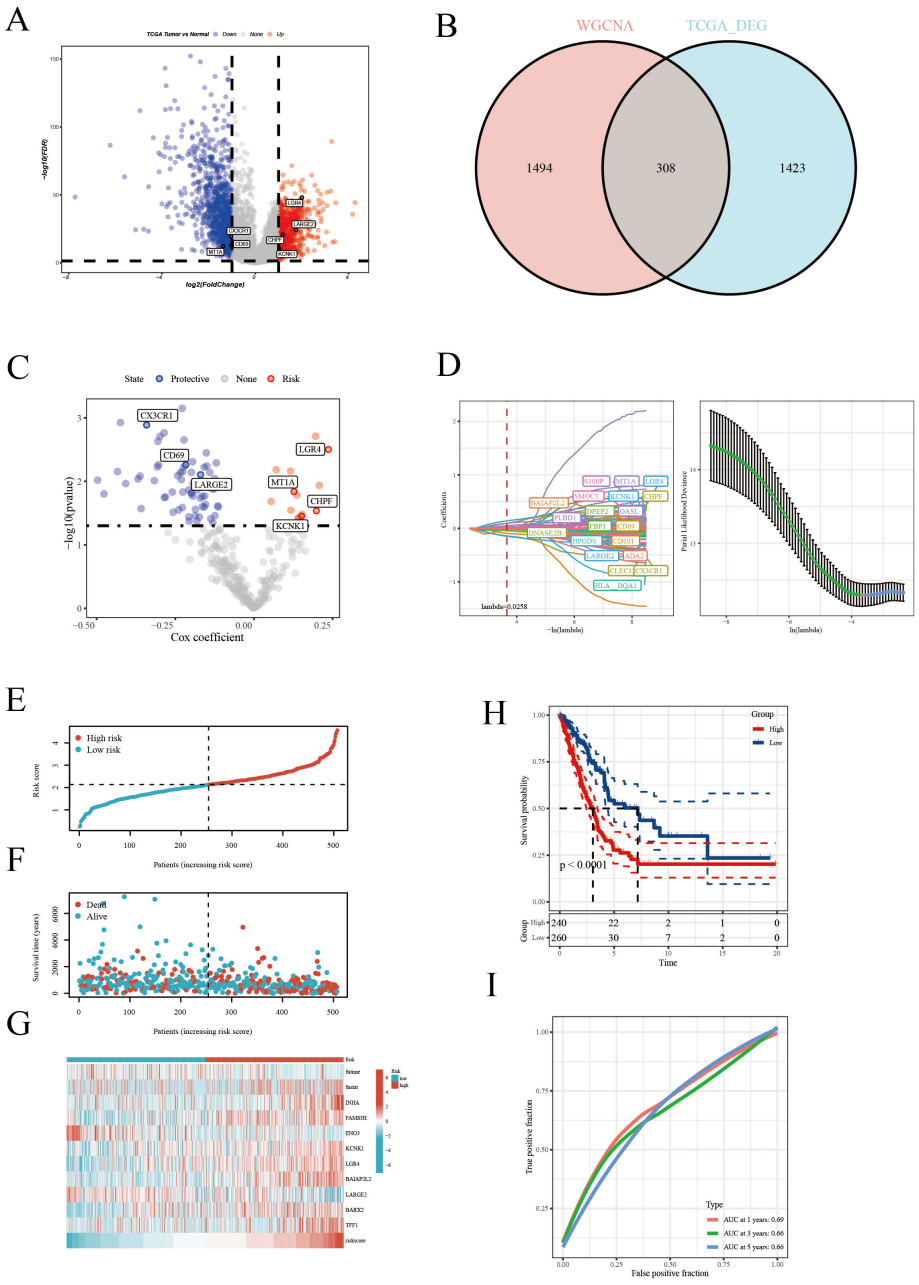
Construction of the prognostic model. **(A)** Volcano plot highlighting significantly upregulated (red) and downregulated (blue) genes. **(B)** Venn diagram of differential genes and WGCNA results, identifying key genes. **(C)** Univariate Cox regression analysis revealing genes significantly associated with patient survival. **(D)** LASSO regression analysis selecting the most survival-relevant genes. **(E)** Risk score distribution differentiating patient risk levels. **(F)** Survival status distribution across risk score groups. **(G)** Heatmap of HUB gene expression across samples. **(H)** Kaplan-Meier survival curves comparing patient survival between risk groups. **(I)** ROC curve assessing the accuracy of 1-, 3-, and 5-year survival predictions.

In this report, leveraging the TCGA [LUAD] dataset, we initially pinpointed genes exhibiting significant expression disparities, as illustrated in the volcano plots (**Figure 3A**). The selection criteria were based on |logFC| ≥ 1 and p < 0.05. Subsequently, integrating insights from the WGCNA analysis, we pinpointed key intersecting genes (**Figure 3B**). These genes were subjected to univariate Cox regression analysis, which demonstrated their significant association with survival outcomes in TCGA [LUAD] samples (**Figure 3C**). Further, employing the LASSO regression method (**Figure 3D**), we narrowed down to 20 crucial genes under a penalty strength of λ=0.0258. Utilizing this refined gene set, we constructed a prognostic scoring model for disulfidptosis related attributes, employing a combined multivariate Cox regression analysis. The risk formula was established as: ‘0.12*KCNK1 +* 0.275*LGR4* - 0.224*CD69* - 0.326*CX3CR1* - 0.247*LARGE2 +* 0.158*MT1A* + 0.206*CHPF’. The risk score successfully categorized patients into high-risk and low-risk groups, as evidenced by the distribution diagram of the risk scores (**Figure 3E**). Concurrently, the survival status distribution diagram (**Figure 3F**) vividly depicted the survival outcomes in both risk categories. Complementary to this, expression heat maps of these HUB genes across different samples (**Figure 3G**) reinforced the rationale behind the stratification based on the median value. Kaplan-Meier survival analyses further corroborated that patients classified within the high-risk group exhibited a significantly poorer prognosis in comparison to their low-risk counterparts (**Figure 3H**). The model’s predictive accuracy for 1-year, 3-year, and 5-year survival rates was confirmed through ROC curves (**Figure 3I**). Additionally, the prognostic and diagnostic value of the model was further substantiated across four independent GEO cohorts (**Supplementary Figure 3**). Collectively, these findings underscore the efficacy of our scoring model, centered on disulfidptosis related attributes, as a robust tool for predicting survival outcomes in LUAD patients.

### 3.4 Clinical characteristics and risk assessment analysis

**Figure 4:**
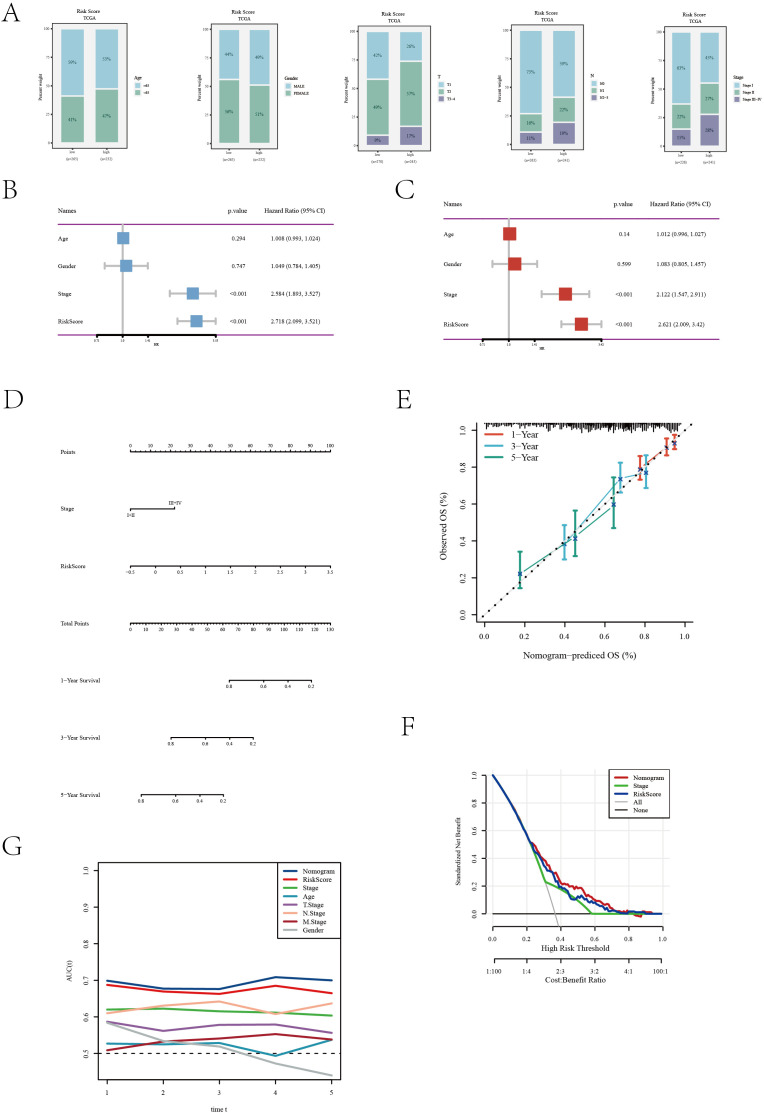
Clinical feature and risk assessment analysis of the TCGA[LUAD] dataset. **(A)** Distribution of various clinical features across different risk groups. **(B)** Univariate Cox regression analysis results showing the association of clinical features with survival outcomes. **(C)** Multivariate Cox regression analysis assessing the independent predictive value of clinical features. **(D)** A nomogram was developed by integrating clinical characteristics with the risk score **(E)** Calibration curve evaluating the calibration of the prediction model. **(F)** Decision curve analysis of the clinical utility of the prediction model. **(G)** ROC curve assessing the accuracy of the prediction model at different time points.

In our further explorations, we scrutinized the efficacy of the disulfidptosis feature scoring model across diverse clinical characteristics, as depicted in **Figure 4A**. The analysis notably demonstrated that the low-risk cohort primarily consisted of younger patients (under 65 years) and a greater proportion of females, whereas the high-risk cohort was predominantly composed of patients in advanced pathological stages. A critical finding from the univariate Cox regression analysis was the identification of pathological staging and risk scoring as significant prognostic indicators for patients with LUAD (**Figure 4B**). This finding was corroborated by multivariate Cox regression analysis, which reinforced the independent prognostic significance of both pathological staging and risk scoring (**Figure 4C**). To visually delineate the impact of these variables on patient prognosis, we constructed a forest plot (**Figure 4D**). This plot concisely illustrated the relative influence of pathological staging and risk scoring on patient outcomes. The calibration curve (**Figure 4E**) further illustrated the model’s high predictive accuracy, closely aligning with the ideal 45-degree line. Decision curve analysis (**Figure 4F**) provided additional evidence of the model’s significant clinical utility across various decision-making thresholds. Moreover, the accuracy of the model in predicting survival rates at 1, 3, and 5 years was evaluated through ROC curves (**Figure 4G**). The area under the curve (AUC) results underscored the model’s robust predictive performance at these distinct time points, thereby affirming the model’s comprehensive efficacy. In summary, our disulfide death feature scoring model emerges as a potent and reliable tool for risk stratification and informing clinical decision-making in the context of LUAD patients.

### 3.5 Enrichment analysis

**Figure 5:**
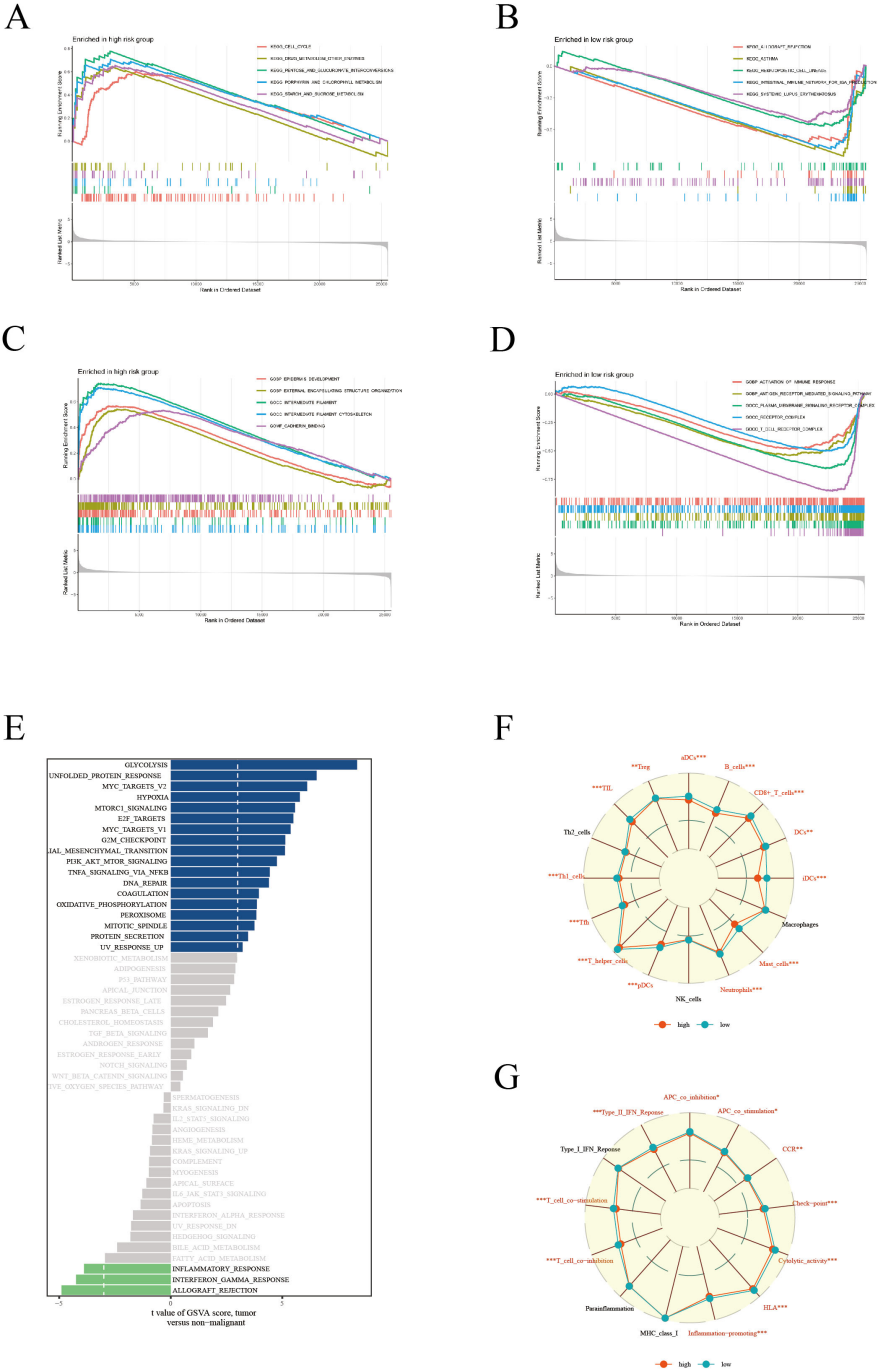
Enrichment analysis. KEGG enrichment analysis showing significant metabolic pathways in high **(A)** and low **(B)** risk groups. GO enrichment analysis revealing cellular processes, molecular functions, and biological processes in high **(C)** and low **(D)** risk groups. **(E)** GSVA results presenting gene set variations across different risk score groups. **(F)** Comparison of immune cell composition between high and low risk groups. **(G)** Comparison of immune function states, highlighting functional activity differences between the two risk groups.

In our comprehensive biological characteristic analysis of patients categorized into high and low-risk groups, significant disparities were observed. The high-risk group demonstrated a predominant enrichment in key KEGG pathways such as Cell Cycle and Drug Metabolism-Other Enzymes (**Figure 5A**). This suggests a potential inclination towards more aggressive cancer progression in this cohort. The low-risk group demonstrated significant enrichment in immune response pathways, specifically Allograft Rejection and Hematopoietic Cell Lineage (**Figure 5B**), hinting at a more robust immune system engagement. Further delving into the Gene Ontology (GO) analysis, the high-risk group revealed significant enrichment in pathways related to Epidermis Development and External Encapsulating Structure Organization (**Figure 5C**). Instead, the low-risk group displayed a marked enrichment in pathways pivotal for immune activation, including Activation of Immune Response and Antigen Receptor-Mediated Signaling Pathway (**Figure 5D**). This dichotomy underscores the distinct biological underpinnings between the two risk groups. GSVA further elucidated that pathways such as Glycolysis and Unfolded Protein Response were particularly active in patients within the high-risk group (**Figure 5E**), indicating a potential link with metabolic stress and cancer aggressiveness. In terms of immune cell infiltration and function, the low-risk group exhibited significantly higher levels of various immune cells, including aDCs, B cells, CD8^+^ T cells, among others (**Figure 5F**).Correspondingly, this group also showed heightened activity in several immune functions like APC co-inhibition, CCR, and type II interferon response (**Figure 5G**), suggesting a more active and potentially effective immune surveillance against tumor cells. Overall, these findings not only elucidate the importance of our risk scoring model in predicting LUAD survival prognosis, but also reveal profound differences in molecular pathways and immune microenvironment between high-risk and low-risk groups. The pronounced immune activity in the low-risk group could be a key factor contributing to a more favorable prognosis. These insights significantly enhance the application value of our scoring model, providing critical biomarkers and therapeutic targets for refining future clinical strategies in LUAD management.

### 3.6 Mutation analysis

**Figure 6:**
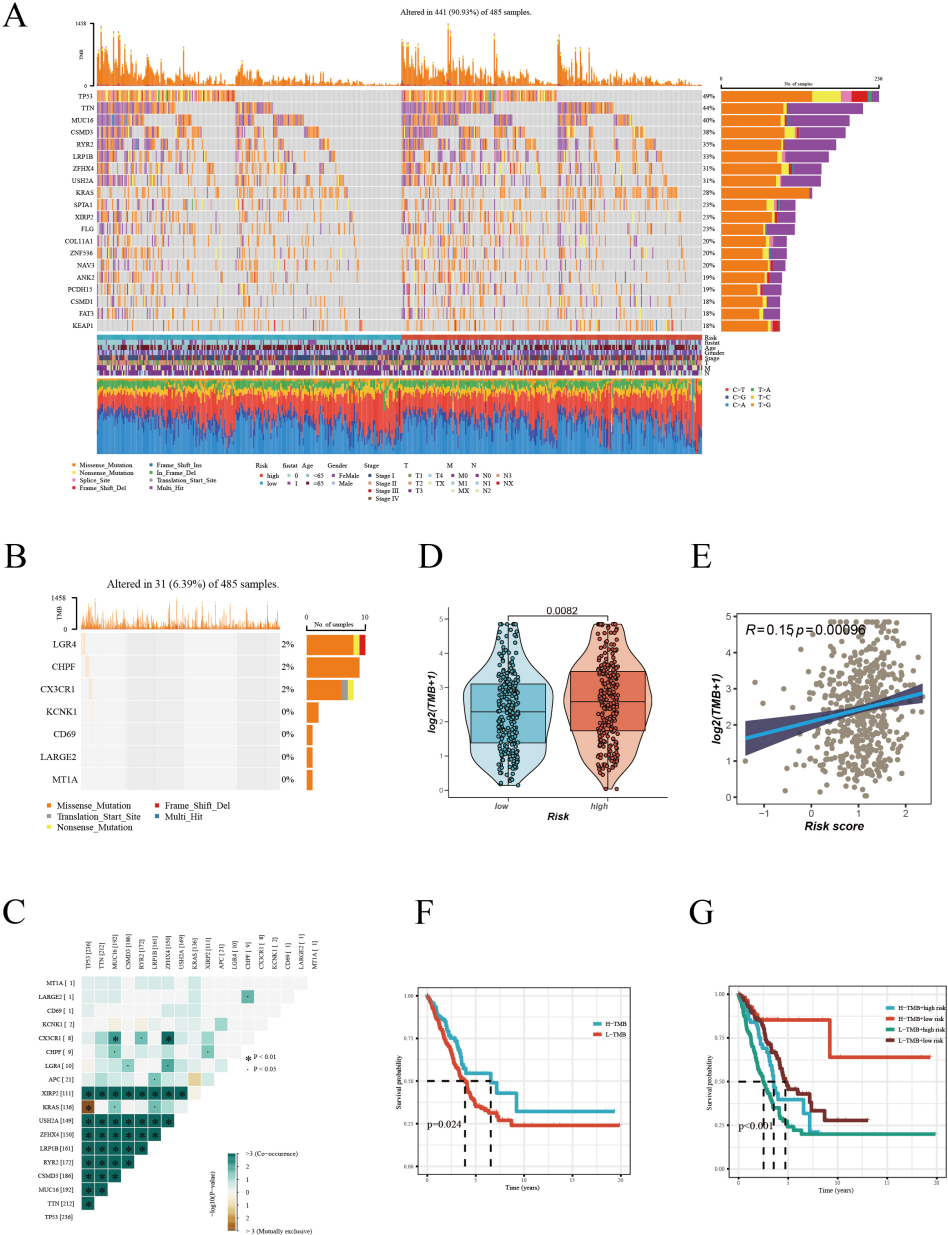
Mutation landscape analysis in LUAD. **(A)** Mutation landscape of high and low risk groups, displaying the top 20 most frequently mutated genes in the TCGA-LUAD dataset. **(B)** Mutation frequency of Hub genes. **(C)** Co-mutation patterns of Hub genes with high-frequency mutated genes. **(D)** Comparison of Tumor Mutation Burden (TMB) between different risk groups. **(E)** Correlation analysis between risk score and TMB. **(F)** Kaplan-Meier survival curves based on high and low TMB. **(G)** Multivariate Kaplan-Meier survival curves combining TMB and risk score.

Our detailed mutation analysis of the TCGA [LUAD] dataset thoroughly mapped the mutation landscape in LUAD patients. The analysis revealed the top 20 genes which were most frequently mutated and their associated clinical information for both high and low-risk groups (**Figure 6A**). Within the mutation profiles of Hub genes, different colors were used to represent various mutation types, with deletions emerging as the predominant mutation type (**Figure 6B**). Further delving into the intricate co-mutation patterns, we examined the relationships between Hub genes and other highly mutated genes. These interconnections were visually depicted in a matrix plot, where darker squares indicated a higher frequency of co-mutations (**Figure 6C**). For example, notable co-mutation relationships were observed between CX3CR1 and genes such as MUC16, RYR2, ZFHX4. The violin plot (**Figure 6D**) succinctly demonstrated that the TMB was significantly higher in patients within the high-risk group, suggesting TMB’s potential role as a crucial biomarker in this subset of LUAD patients. Moreover, a positive correlation was established between the risk score and TMB (**Figure 6E**), indicating a close link between increased mutation load and the prognostic risk score in LUAD. Kaplan-Meier survival curves (**Figure 6F**) traditionally indicated better survival prognosis in patients with higher TMB. However, a compelling observation emerged when TMB was considered alongside risk scoring (**Figure 6G**): although high TMB is typically viewed as a favorable prognostic factor, in patients with high-risk scores, low TMB correlated with poorer survival outcomes. This nuanced finding underscores the complexity of interpreting TMB in the context of LUAD and highlights the necessity of integrating TMB with risk scoring for a holistic assessment of patient prognosis. These insights are pivotal for LUAD management, particularly in tailoring treatment strategies for high-risk patients. Our study underscores the potential of precision medicine in treating LUAD, advocating for customized treatment plans based on detailed genetic mutation analysis in tandem with individual clinical characteristics to enhance patient survival prognosis.

### 3.7 Tumor microenvironment assessment

**Figure 7:**
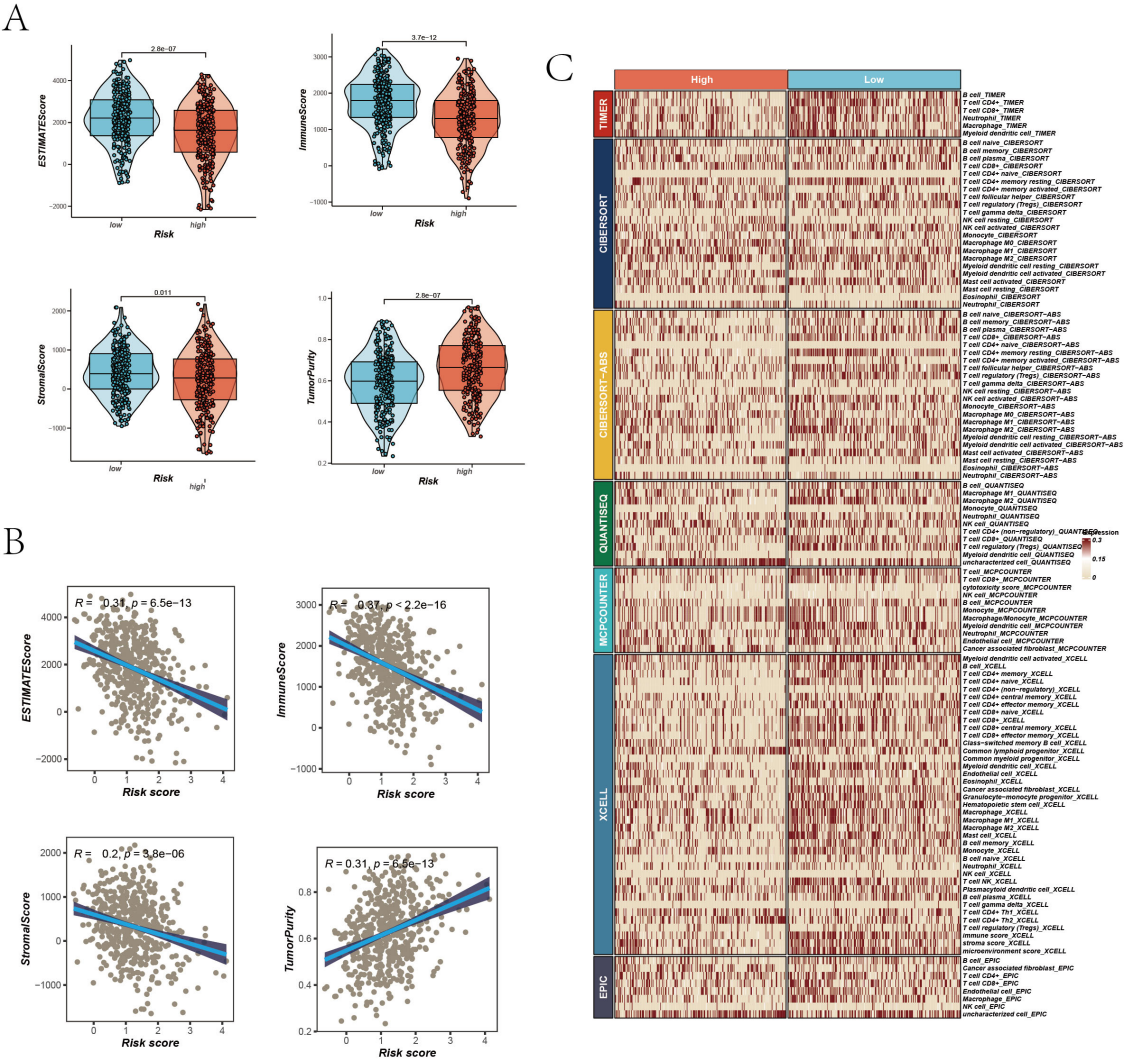
TME Assessment. **(A)** Differences in ESTIMATE Score, Immune Score, Stromal Score, and Tumor Purity between high and low-risk groups, represented via violin plots with boxplots indicating distributions and median values. **(B)** Scatter plots correlating risk scores with ESTIMATE Score, Immune Score, Stromal Score, and Tumor Purity, each with correlation coefficients (R²) and P values. **(C)** Immune infiltration assessment in high and low-risk groups across seven algorithms, visualized in a heatmap displaying the relative abundance of various immune cell types.

In our in-depth study exploring the complex relationship between TME characteristics and patient risk scores, we unearthed several significant insights. Notably, the analysis demonstrated that in the low-risk group, key parameters such as the ESTIMATE Score, Immune Score, and Stromal Score were considerably elevated compared to the high-risk group. This finding indicates a more robust immune and stromal component presence within the TME of patients classified as low-risk group (**Figure 7A**). Conversely, the high-risk group exhibited increased tumor purity, suggesting a reduced presence of non-tumor components in their TME (**Figure 7B**). Additionally, a pronounced negative correlation was observed between the risk score and scores indicative of immune and stromal components (ESTIMATE Score, Immune Score, and Stromal Score). Conversely, a positive correlation emerged with Tumor Purity, elucidating a trend where the proportion of immune and stromal components diminishes with an increasing risk score. To validate these findings, we used the Timer 2.0 database to compare immune infiltration levels between high and low-risk groups. The results highlighted that the low-risk group exhibited a higher level of immune cell infiltration, as shown in the heatmaps (**Figure 7C**). In conclusion, our findings reveal that tumors in the high-risk group exhibit greater purity, whereas those in the low-risk group are characterized by a more pronounced infiltration of immune cells.

### 3.8 Evaluation and validation of immunotherapy efficacy

**Figure 8:**
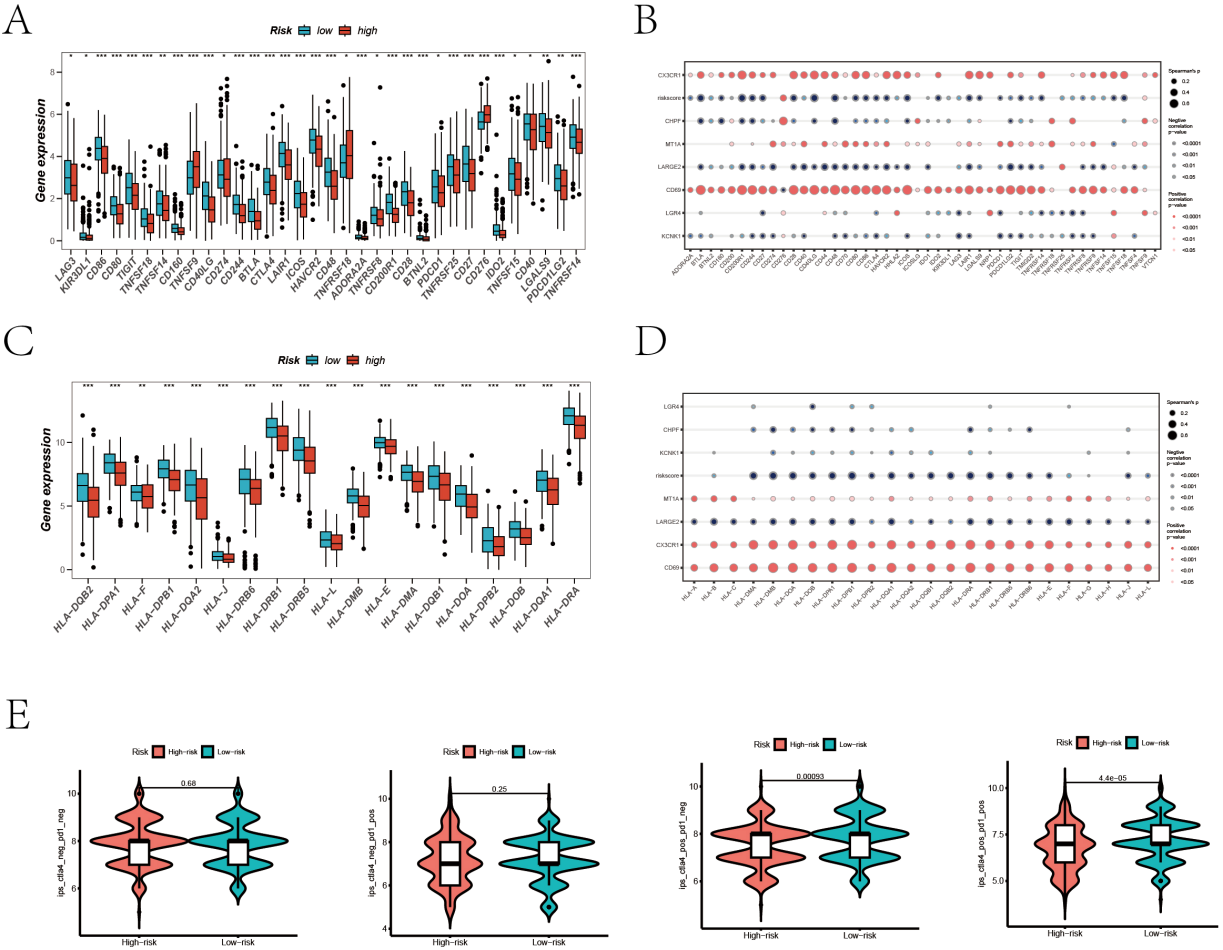
Prediction of Immunotherapy Efficacy. **(A)** Differences in immune checkpoint gene expression between high and low-risk groups. **(B)** Correlations between immune checkpoint gene expression, risk scores, and Hub genes. **(C)** Expression differences of major histocompatibility complex (MHC) genes between risk groups. **(D)** Correlations of MHC-related genes and immune checkpoint genes with risk scores and Hub genes. **(E)** Comparative Immune Predictive Scores (IPS) between high and low-risk groups.

Given the significant progress in immunotherapy for LUAD, our study conducted a comprehensive assessment of the responsiveness of different patient risk groups to immunotherapy using various methodologies. Inspired by previous studies, as cited in references, which reported that high expression levels of immune checkpoint related and Major Histocompatibility Complex (MHC) related genes could predict a better response to immunotherapy, we explored the levels of these genes in various risk groups of LUAD patients in our study (35–37). **Figures 8A–D** show a detailed analysis of immune checkpoint and MHC gene expression differences between high and low-risk groups, revealing significant variations. Additionally, we examined the association between the expression of model genes, risk stratification, and the specified gene categories. The analysis found that key immunotherapy-related genes were expressed at higher levels in the low-risk group versus the high-risk group. Furthermore, there was a significant negative correlation between the risk score and the expression of these gene categories. To further substantiate our findings, we compared the Immune Phenotype Scores (IPS) (**Figure 8E**), revealing that in LUAD patients positive for CTLA4 and negative for PD-L1, as well as those positive for both CTLA4 and PD-L1, patients in the low-risk cohort showed higher IPS scores.

This suggests a greater likelihood of benefiting from immunotherapy in these patients.

To enhance the credibility of our analysis, we extended our validation to two public immunotherapy datasets, IMvigor210 and GSE78220. The survival analysis of the IMvigor210 dataset reveals a poor prognosis for high-risk cohorts across the entire patient population, as well as within both early-stage and late-stage subgroups (**Supplementary Figures 4A–C**). Notably, patients with better therapeutic responses (Complete Response/Partial Response, CR/PR) exhibited significantly lower risk scores (**Supplementary Figure 4D**), and a higher proportion of CR/PR was observed in the low-risk cohort. Consistent results were obtained from the GSE78220 dataset (**Supplementary Figures 4F–H**). Collectively, this study demonstrates that LUAD patients in the low-risk cohort, characterized by high expression of immune checkpoint and MHC related genes, are more predisposed to benefit from immunotherapy.

### 3.9 Basic experiments explore the role of KCNK1 in LUAD

**Figure 9:**
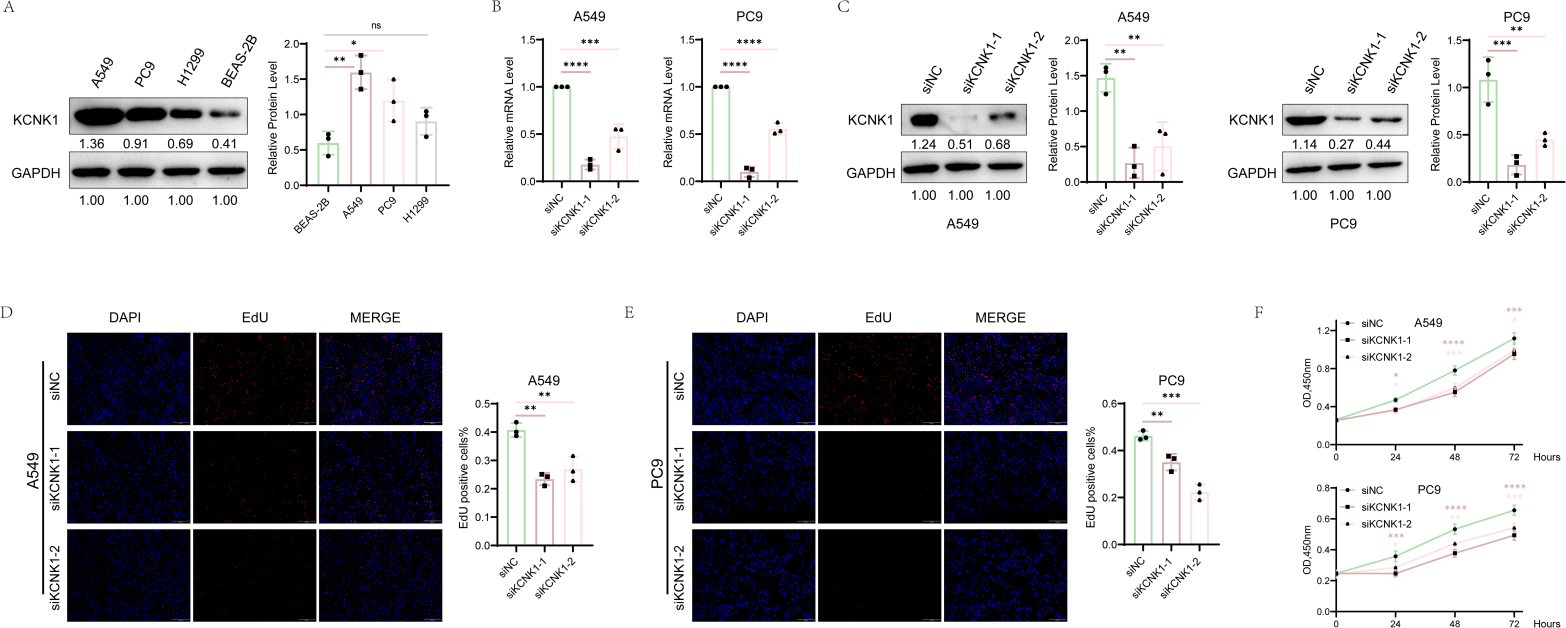
The Correlation Between KCNK1 Expression and Proliferative Capacity in LUAD Cells. **(A)** Comparative analysis of KCNK1 protein expression levels between normal bronchial epithelial cells and LUAD cell lines. **(B, C)** The efficiency of KCNK1 knockdown was assessed using RT-qPCR and Western blot. **(D, E)** EdU assay was employed to evaluate the impact of KCNK1 on the proliferation of LUAD cells, with the proliferation index (PI) calculated as PI = number of red dots (cells in the proliferative phase)/total number of blue dots (total cells). **(F)** CCK-8 assay measured cell absorbance at 450nm over 24, 48, and 72 hours, respectively. *P < 0.05, **P < 0.01, ***P < 0.001, ****P < 0.0001.

**Figure 10:**
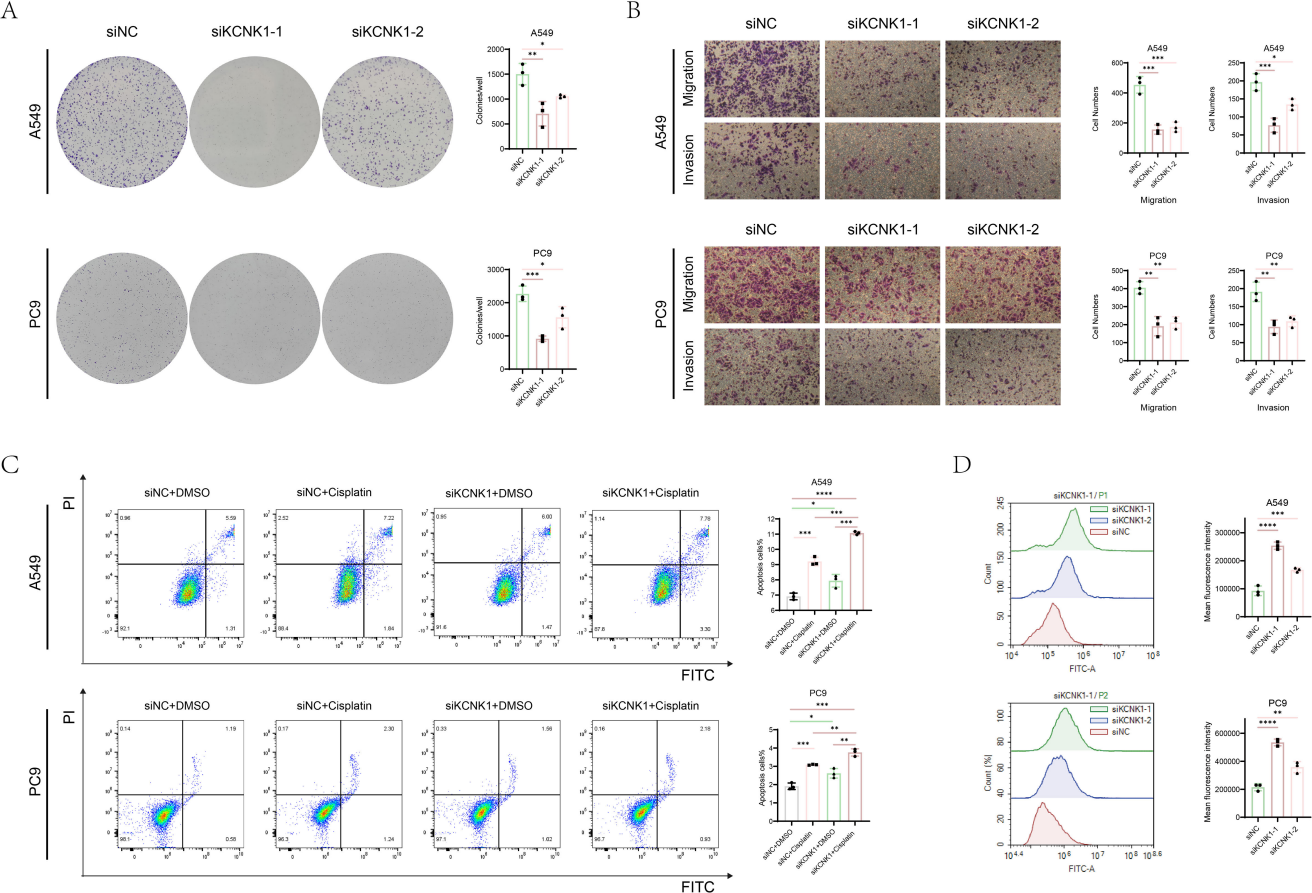
The function of KCNK1 in LUAD. **(A)** Colony formation assays were used to explore the cell colony formation ability of A549 and PC9 cells. **(B)** The migration and invasion abilities of A549 and PC9 cells. **(C)** Flow cytometry was used to detect apoptosis rate of A549 and PC9 cells which were transfected with siNC or siKCNK1 and received either Cisplatin or DMSO treatment. **(D)** ROS levels in A549 and PC9 cells transfected with siNC and siKCNK1 was assessed by flow cytometry. *P < 0.05, **P < 0.01, ***P < 0.001, ****P < 0.0001.

Review of previous literature indicated that KCNK1 (Potassium Two Pore Domain Channel Subfamily K Member 1), a crucial member of the potassium channel family, is highly expressed in various cancers and significantly related to tumor prognosis and invasiveness (38). However, its role in LUAD has not been fully elucidated, guiding us to design tests to explore its function. We began by assessing KCNK1 expression in lung epithelial cells (BEAS-2B) and LUAD cell lines (A549, PC9, H1299) through western blot, revealing its significant overexpression in LUAD (**Figure 9A**). We then developed specific siRNAs targeting KCNK1 and confirmed their efficiency in reducing KCNK1 expression in A549 and PC9 cells using RT-qPCR and western blot (**Figures 9B, C**). Subsequent EdU and CCK-8 assays demonstrated that KCNK1 siRNA transfection significantly reduced LUAD cell proliferation (**Figures 9D–F**), and colony formation assays showed decreased colony-forming abilities in these cells (**Figure 10A**). Transwell assays further indicated that KCNK1 knockdown markedly reduced cell migration and invasion (**Figure 10B**). Investigating the link between KCNK1 and chemotherapy resistance, particularly to cisplatin, a key LUAD treatment drug, we found that KCNK1 downregulation and cisplatin treatment synergistically promoted cell apoptosis (**Figure 10C**), suggesting KCNK1’s association with cisplatin resistance in LUAD. Lastly, we examined the effect of KCNK1 knockdown on ROS levels, finding increased ROS production in the knockdown group (**Figure 10D**), implying that KCNK1 inhibition might enhance disulfidptosis by elevating ROS production.

## 4 Discussion

Redox homeostasis is essential for the sustenance of cellular life. In comparison to normal tissues, cancer cells frequently experience elevated oxidative stress as a result of genetic mutations and metabolic reprogramming (39, 40). This pathological state induces mitochondrial dysfunction through electron transport chain impairment, resulting in excessive reactive oxygen species (ROS) production alongside compensatory activation of antioxidant defenses such as glutathione (GSH) and superoxide dismutase (SOD) to ensure survival. A biphasic ROS response emerges: Subtoxic levels activate proto-oncogenic signaling pathways (e.g., NF-κB, AP-1) to drive tumor progression; Supraphysiological accumulation causes oxidative DNA damage-induced genomic instability and initiates programmed cell death pathways, including apoptosis, ferroptosis, and disulfidptosis. Glutathione (GSH) plays a crucial role in neutralizing excess ROS, necessitating that cancer cells maintain sufficient GSH levels to ensure survival and proliferation (41, 42). Disulfidptosis represents a distinct form of programmed cell death (PCD) triggered by oxidative stress, characterized by the excessive formation of disulfide bonds (-S-S-) in cysteine residues under aberrant oxidative conditions (43). This process leads to protein misfolding, functional loss, and ultimately, cell death. The induction of disulfidptosis may require several critical factors, including increased ROS production, upregulation of SLC7A11 expression, glucose deprivation, and abnormal disulfide bond formation in actin filaments (13, 44–46). An increase in ROS directly contributes to the formation of abnormal protein disulfide bonds. Under normal conditions, protein disulfide bonds are primarily formed in the oxidizing environment of the endoplasmic reticulum (47). Oxidative stress can facilitate the formation of protein disulfide bonds within the cytoplasm, which is typically a reducing environment (48–50). In such conditions, proteins frequently develop aberrant disulfide bonds. SLC7A11, a critical amino acid transporter, is upregulated to enhance the cystine/glutamate exchange process. Upon the transport of cystine into the cytoplasm by SLC7A11, it is imperative for the cell to rapidly convert cystine into the more soluble cysteine to facilitate glutathione synthesis (51, 52). This conversion necessitates NADPH, which is generated by the oxidative pentose phosphate pathway, serving as an essential reducing agent. In scenarios of glucose metabolism dysregulation or glucose deprivation, the cellular supply of NADPH is compromised, resulting in the abnormal accumulation of cystine and other disulfide-linked molecules, thereby inducing disulfide stress. Consequently, aberrant disulfide bonds form within actin cytoskeletal proteins, leading to the collapse of the actin network and the phenomenon known as disulfidptosis (53, 54).

Disulfidptosis exerts multifaceted impacts on tumor cell chemoresistance, senescence, and neurodegenerative pathogenesis. Li M et al. developed a risk prediction model based on disulfidptosis-related genes (DRGs) in colon adenocarcinoma patients, demonstrating that POU4F1 knockdown (a DRG) markedly attenuated COAD cell proliferation, migration, and disulfidptosis susceptibility while augmenting cellular senescence (55, 56).Notably, hepatocellular carcinoma (HCC) patients with elevated disulfidptosis activity exhibited enhanced therapeutic responsiveness to PD-1/PD-L1 blockades (55, 56). Furthermore, Alzheimer’s disease (AD) studies have identified that enriched pathways of disulfidptosis-associated differentially expressed genes (DEGs) critically regulate neurological homeostasis, highlighting their potential involvement in AD-related pathological cascades (57). Collectively, these findings position disulfidptosis as a promising therapeutic target spanning oncological and neurodegenerative disorders.

LUAD, the most common subtype of lung cancer, comprises 40% of cases. The treatment options for LUAD are diverse, encompassing surgical intervention primarily for early-stage disease, radiotherapy for locally advanced cases or as adjuvant therapy post-surgery, and chemotherapy, particularly for advanced or metastatic LUAD (43, 58). Platinum-based chemotherapy, with Cisplatin as a key agent, remains a cornerstone in the therapeutic management of lung adenocarcinoma. Nevertheless, the prognosis for LUAD patients remains poor due to rapid tumor progression and the emergence of resistance to existing therapies, resulting in a five-year survival rate of merely 15%. Consequently, investigating the regulatory mechanisms underlying tumor growth and treatment efficacy has become increasingly critical.

In the realm of LUAD, metabolic reprogramming plays a pivotal role, not only in facilitating the rapid growth and survival of tumor cells but also in potentially elevating intracellular reactive oxygen species (ROS) levels, as noted in (59). This increase in ROS may trigger disulfide death, significantly impacting LUAD cell survival. Furthermore, preclinical findings have illustrated that metabolic intervention, specifically via glucose transporter (GLUT) inhibitors, can initiate disulfide death, thereby impeding renal cancer cell proliferation. Delving into disulfide death in LUAD offers critical insights into how alterations in tumor cell metabolism influence their behavior. It also lays the groundwork for novel therapeutic approaches, targeting these metabolic pathways. This might include strategies focused on ROS regulation and GSH metabolism, presenting new avenues for LUAD treatment advancement. Consequently, disulfide death-related genes emerge as promising biomarkers or therapeutic targets, potentially playing a transformative role in enhancing LUAD treatment outcomes.

The prognostic model incorporates multiple genes, with BAIAP2L2 being upregulated in LUAD tissue, where its elevated expression is positively associated with poor prognosis (60). Similarly, the upregulation of FAM83H-AS1 is indicative of an unfavorable prognosis for LUAD patients (61). Increased expression of INHA in LUAD tissue correlates with reduced OS and more advanced pathological stages. Furthermore, INHA expression is linked to immune cell infiltration and immune-related markers within the LUAD TME (62). LGR4 is significantly overexpressed in LUAD, and endogenous RSPO3-LGR4 signaling not only enhances cell migration and invasion but also facilitates the epithelial-mesenchymal transition (EMT) in LUAD cells (63, 64). BARX2 is notably upregulated in LUAD tissues and is positively correlated with advanced clinical stages and poor prognosis (65). Additionally, aberrant expression of KCNK1 is associated with the malignancy of various cancers.

For example, the expression of this marker in thyroid cancer correlates with clinical staging and is upregulated in hepatocellular carcinoma, where it functions as a potential prognostic and diagnostic biomarker (66, 67). In a similar vein, KCNK1 is overexpressed in breast cancer due to promoter hypomethylation, which is linked to poor prognosis and suboptimal treatment response (68). Survival analysis of the proposed prognostic model indicated that individuals in the high-risk group had a poorer prognosis, a finding that was validated across four independent GEO cohorts.

Moreover, by integrating clinical traits with risk scores, we developed an innovative nomogram. This nomogram enhances the accuracy of survival predictions for patients with LUAD, serving as a valuable tool for risk stratification and supporting clinical decision-making processes.

The TME, a crucial regulator of tumor growth and metastasis, is characterized by intricate interactions between tumor cells and various non-tumor entities, including immune and stromal cells, vascular endothelial cells, and the extracellular matrix (ECM). These interactions profoundly influence tumor development, invasiveness, and treatment responsiveness (69). Immune cells within the TME exhibit a dual role: while they can suppress tumor growth, they are also susceptible to manipulation by tumor cells to facilitate immune escape (70). The hypoxic conditions and metabolic shifts in the TME critically impact drug sensitivity and efficacy (71). Recent advances in targeting the TME, notably immune checkpoint inhibitors, have underscored its significance in cancer therapy (72, 73). Our study reveals that patients in the low-risk group exhibit stronger immunogenicity and greater immune cell infiltration, indicating a more active immune microenvironment, which might respond more favorably to immune-enhancing interventions like checkpoint inhibitors. Conversely, the high-risk group’s tumor purity, reflecting lower immune infiltration, suggests potential immune evasion by tumor cells, necessitating strategies to modify the TME and boost immune response. Lower risk scores were found to be linked to improved response to immunotherapy in the IMvigor210 and GSE78220 datasets, align with this observation. This underscores the importance of personalized treatment approaches and distinct strategies for different risk groups in LUAD. Immunotherapy using immune checkpoint inhibitors has made significant progress in the treatment of Gastric cancer (GC). Therapeutic antibodies targeting the programmed cell death protein-1 (PD-1)/programmed cell death ligand 1 (PD-L1) pathway have been effectively used in the clinical treatment of cancer. Our findings on the differences in immune checkpoint expression among risk groups may establish a molecular foundation for the optimization of PD-1/PD-L1 inhibitor selection in clinical practice (74).

KCNK1, also referred to as TWIK-1 (75), is a member of the two-pore domain potassium channel (K2P) family. These channels are characterized by two transmembrane domains, or helices, and typically function as dimers within the cell membrane to achieve functional expression. Recently, the pivotal role of K2P channels in malignancies has garnered increasing attention (76, 77). Notably, the K2P channel member KCNK3 has been demonstrated to activate the AMPK-TXNIP pathway, thereby inhibiting the proliferation and glucose metabolism of LUAD (78). Despite the involvement of KCNK1 in various cancers, as documented in previous studies, its role in LUAD has not been reported. In this study, we have identified KCNK1 as a potential oncogenic biomarker for LUAD, highlighting its significant role in tumor progression and chemotherapy resistance. Specifically, KCNK1 was identified as a key gene within this signature, showing a significant positive correlation with poor patient prognosis. Elevated expression of KCNK1 has been linked to enhanced proliferation, colony formation, and migration/invasion in LUAD cells, underscoring its oncogenic potential. Additionally, we investigated the properties of KCNK1 related to cisplatin resistance and discovered that high KCNK1 expression correlates with increased resistance to cisplatin in LUAD. Silencing KCNK1 expression augmented apoptosis in LUAD cells and significantly improved the efficacy of cisplatin treatment. Our observations revealed that the downregulation of KCNK1 expression markedly elevates reactive oxygen species (ROS) production. This increase in ROS can lead to the oxidation of cysteine residues in proteins, causing a redox imbalance and the formation of aberrant disulfide bonds within the cytoplasm. Such disruptions interfere with the folding and function of cytoskeletal proteins, thereby inducing disulfidptosis. Prior research has demonstrated that SLC7A11 is overexpressed in LUAD, facilitating increased cystine uptake and promoting glutathione synthesis to mitigate oxidative stress (79). Furthermore, abnormal alterations in glucose metabolism represent a hallmark metabolic characteristic of numerous cancer cells (80).

Recent studies have indicated that KCNK3 plays a role in the glucose metabolism of LUAD cells, with its upregulation leading to the downregulation of GLUT1. This interaction may elucidate a potential mechanism through which KCNK1 contributes to cisplatin resistance in LUAD.

While our study highlights KCNK1’s role in cisplatin resistance, whether KCNK1 influences the generation of NADPH in the pentose phosphate pathway by regulating the expression of SLC7A11 and thereby participates in the regulation of disulfidptosis to control LAUD resistance remains to be fully clarified. Recent advances in functional genomics, such as CRISPR-based screening combined with drug sensitivity profiling, have proven powerful in uncovering novel resistance genes. For instance, a study by Liu H et al. demonstrated how CRISPR screening identified key genes driving resistance to trametinib, a MEK inhibitor, providing a methodological blueprint for future investigations into disulfidptosis-associated resistance (81). The PDX model holds the advantage of preserving the genetic and histological traits of the original tumor, thereby offering a more precise portrayal of human cancer biology. Kang Z et al. employed the PDX model to investigate the metabolic alterations in colorectal cancer (82). In the future, we will also undertake research involving patient-derived xenograft (PDX) models to validate the clinical relevance of this study.

In conclusion, this study has established a comprehensive prognostic model for LUAD utilizing DRGs, which demonstrates efficacy in predicting patient outcomes and the effectiveness of immunotherapy. The gene KCNK1 is identified as a pivotal oncogene in LUAD, exerting a substantial influence on tumor progression and resistance to cisplatin. Increased expression of KCNK1 is associated with unfavorable prognoses and disulfidptosis, indicating that therapeutic targeting of KCNK1 may improve the efficacy of cisplatin treatment.

## Data Availability

The original contributions presented in the study are included in the article/**Supplementary Material**. Further inquiries can be directed to the corresponding author/s.

## Supplementary material

Supplementary Figure 1 Quality control and normalization of scRNA-seq data. **(A)** Pre-quality control data presentation. **(B)** Post-quality control data. **(C)** Patient distribution before batch effect correction. **(D)** Post-correction patient distribution. **(E)** Expression of marker genes at the single-cell level.

Supplementary Figure 2 Analysis of Intercellular Communication. **(A)** Variations in the frequency and intensity of intercellular communication between cells exhibiting high versus low disulfidptosis activity. **(B)** Comprehensive data on differentially expressed pathways in cell communication between the two groups. **(C)** Assessment of the activity levels of each cell type in intercellular communication across the different groups.

Supplementary Figure 3 Prognostic and diagnostic performance of the model across multiple datasets. Kaplan-Meier survival and ROC curves demonstrating the model’s performance in datasets GSE3141, GSE31210, GSE5081, and GSE68465.

Supplementary Figure 4 Validation of the prediction model in two independent immunotherapy cohorts (IMvigor210 and GSE78220). **(A–C)** Survival differences between risk groups in the IMvigor210 cohort. **(D)** Risk score distributions across different immunotherapy responses. **(E)** Proportional bar graphs of immunotherapy responses in risk groups. **(F)** Survival differences in the GSE78220 cohort. **(G)** Risk score differences in immunotherapy outcomes. **(H)** Proportional bar graphs of immunotherapy efficacy in risk groups.
