# Supplementary figures and images for: Toward precision oncology in LUAD: a prognostic model using single-cell sequencing and WGCNA based on a disulfidptosis relative gene signature
Source: Front Immunol. 2025 May 21;16:1581915. doi: 10.3389/fimmu.2025.1581915 (PMC12133857; doi:10.3389/fimmu.2025.1581915)

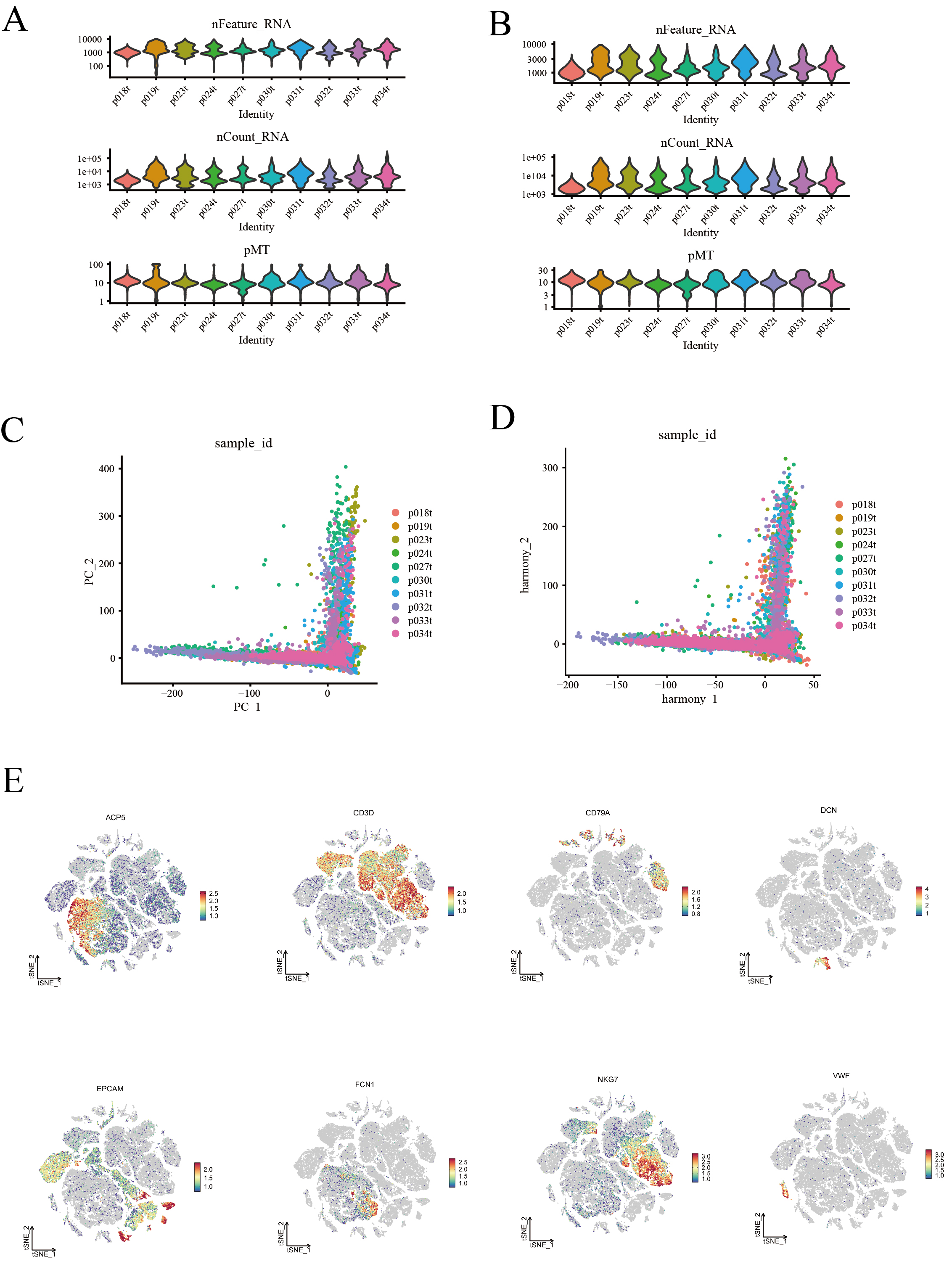

Supplement: Supplementary Figure 1 — Quality control and normalization of scRNA-seq data. (A) Pre-quality control data presentation. (B) Post-quality control data. (C) Patient distribution before batch effect correction. (D) Post-correction patient distribution. (E) Expression of marker genes at the single-cell level. [file Image1.tif]

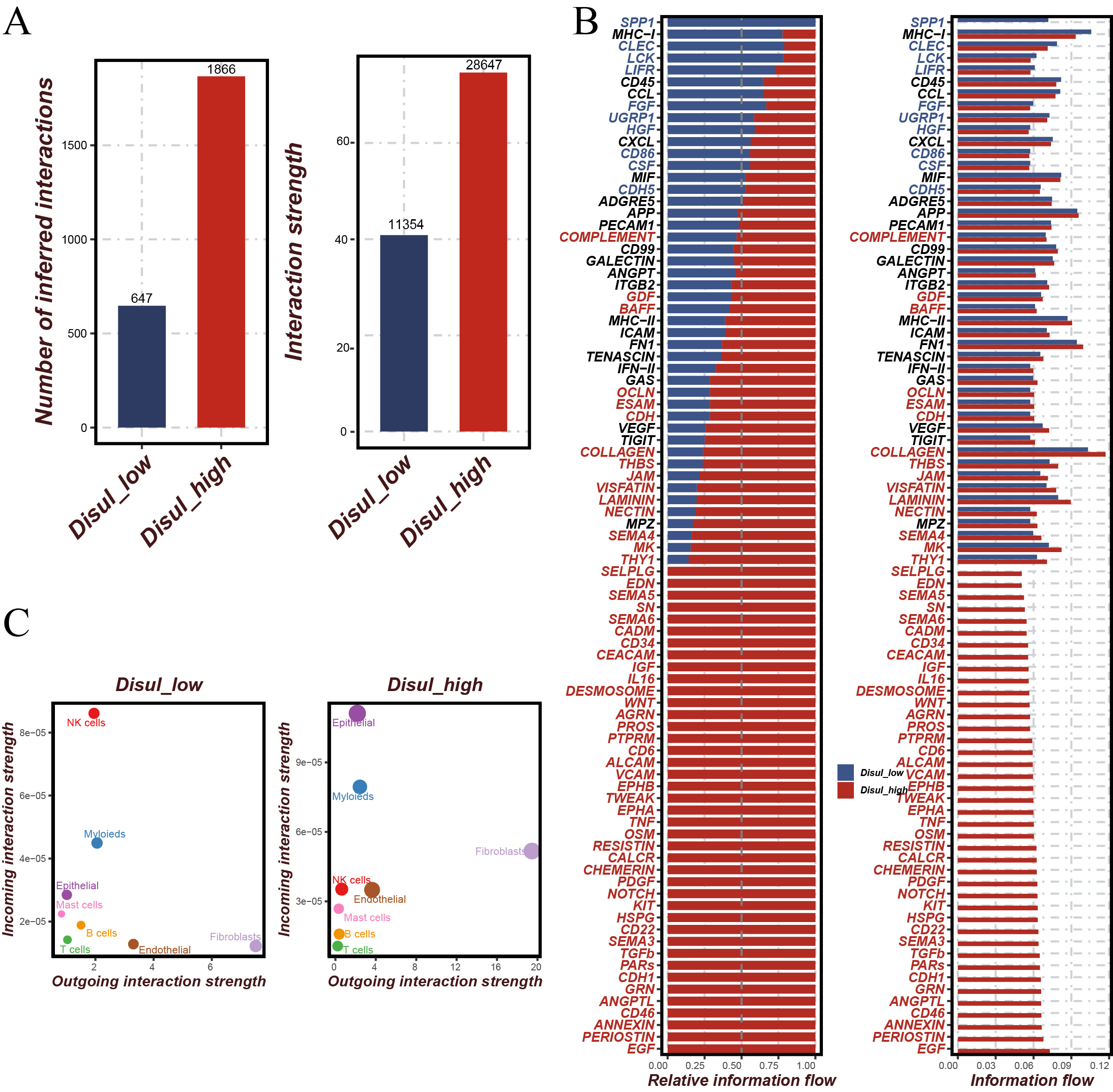

Supplement: Supplementary Figure 2 — Analysis of Intercellular Communication. (A) Variations in the frequency and intensity of intercellular communication between cells exhibiting high versus low disulfidptosis activity. (B) Comprehensive data on differentially expressed pathways in cell communication between the two groups. (C) Assessment of the activity levels of each cell type in intercellular communication across the different groups. [file Image2.tif]

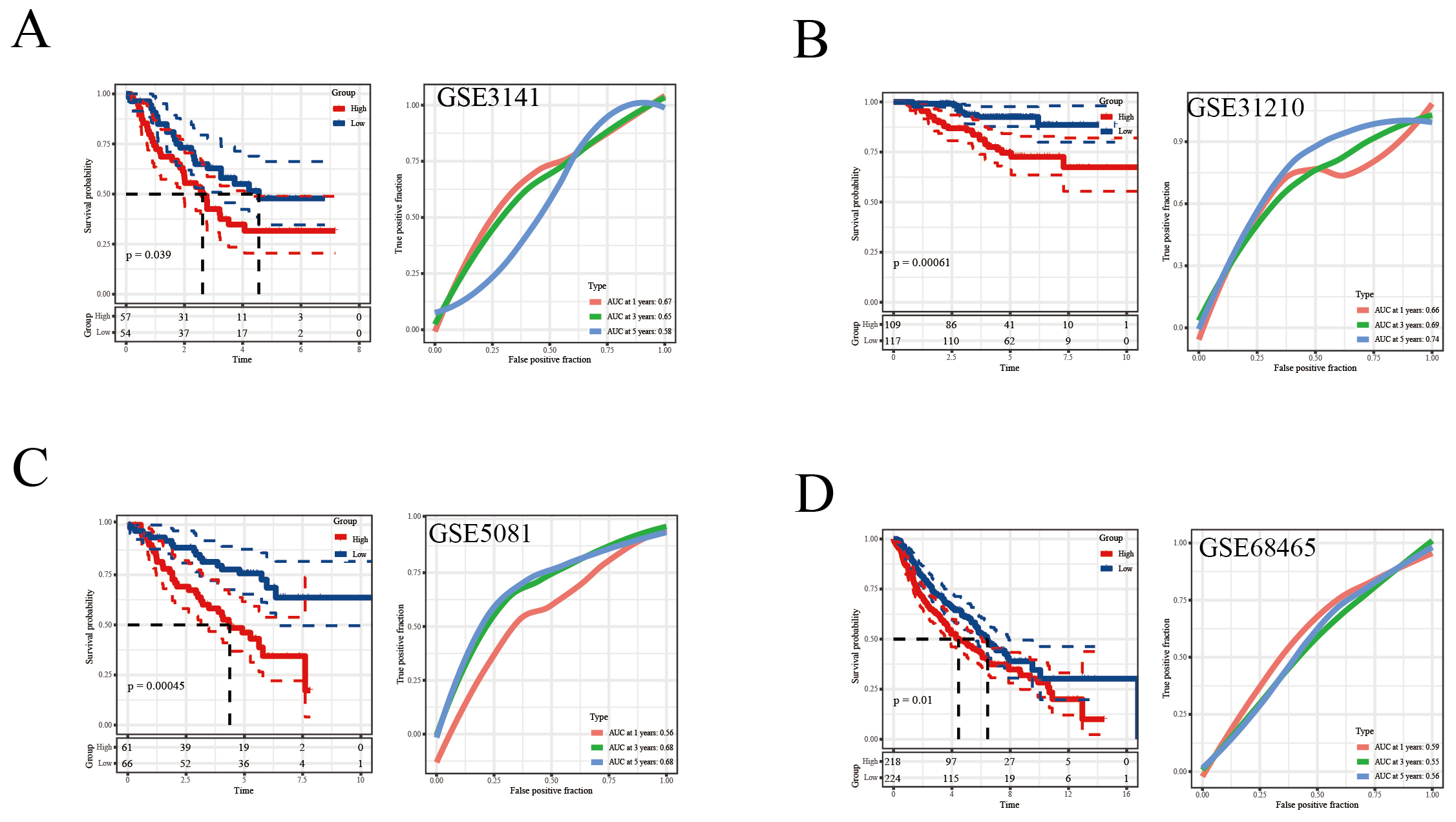

Supplement: Supplementary Figure 3 — Prognostic and diagnostic performance of the model across multiple datasets. Kaplan-Meier survival and ROC curves demonstrating the model’s performance in datasets GSE3141, GSE31210, GSE5081, and GSE68465. [file Image3.tif]

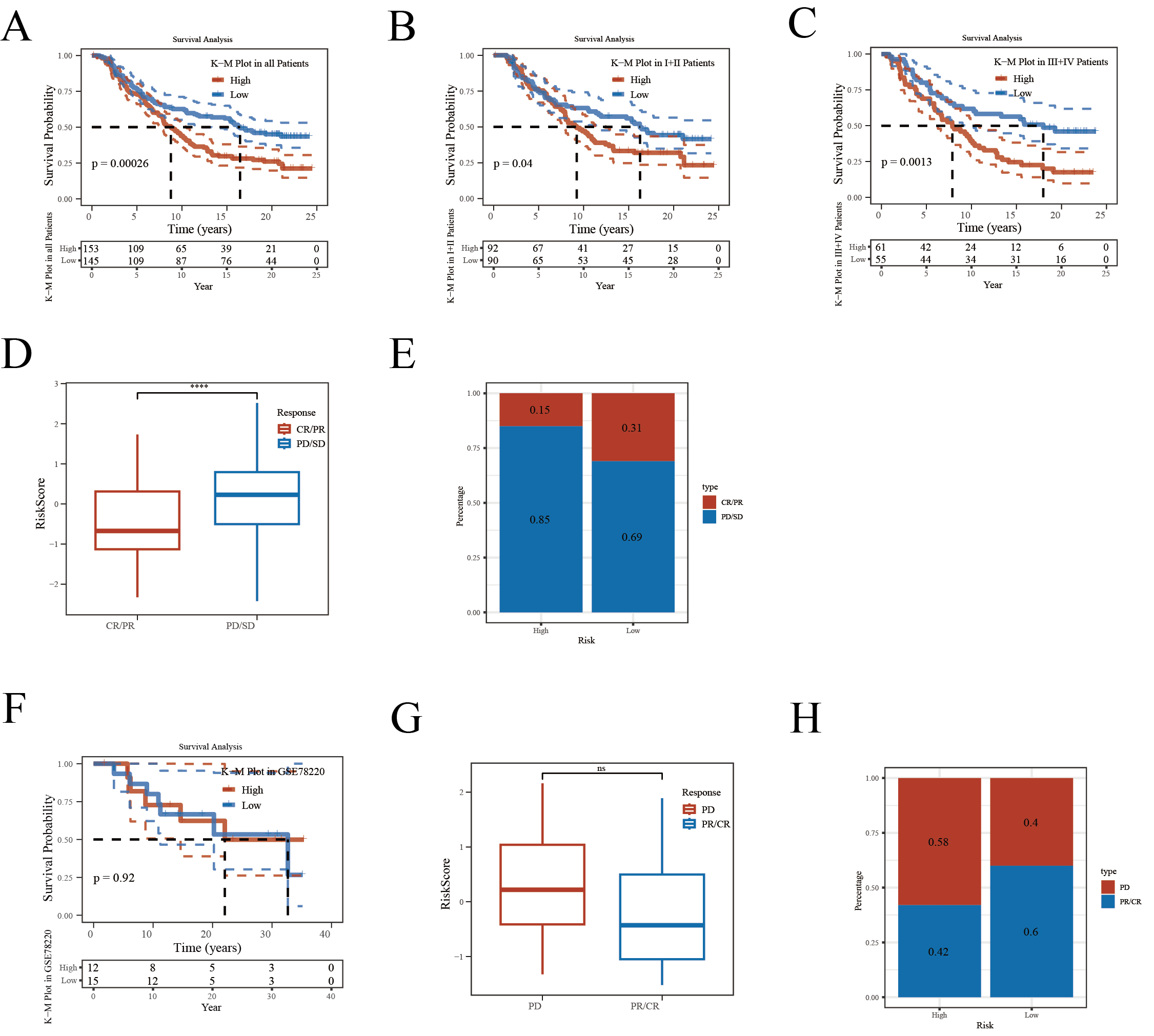

Supplement: Supplementary Figure 4 — Validation of the prediction model in two independent immunotherapy cohorts (IMvigor210 and GSE78220). (A–C) Survival differences between risk groups in the IMvigor210 cohort. (D) Risk score distributions across different immunotherapy responses. (E) Proportional bar graphs of immunotherapy responses in risk groups. (F) Survival differences in the GSE78220 cohort. (G) Risk score differences in immunotherapy outcomes. (H) Proportional bar graphs of immunotherapy efficacy in risk groups. [file Image4.tif]

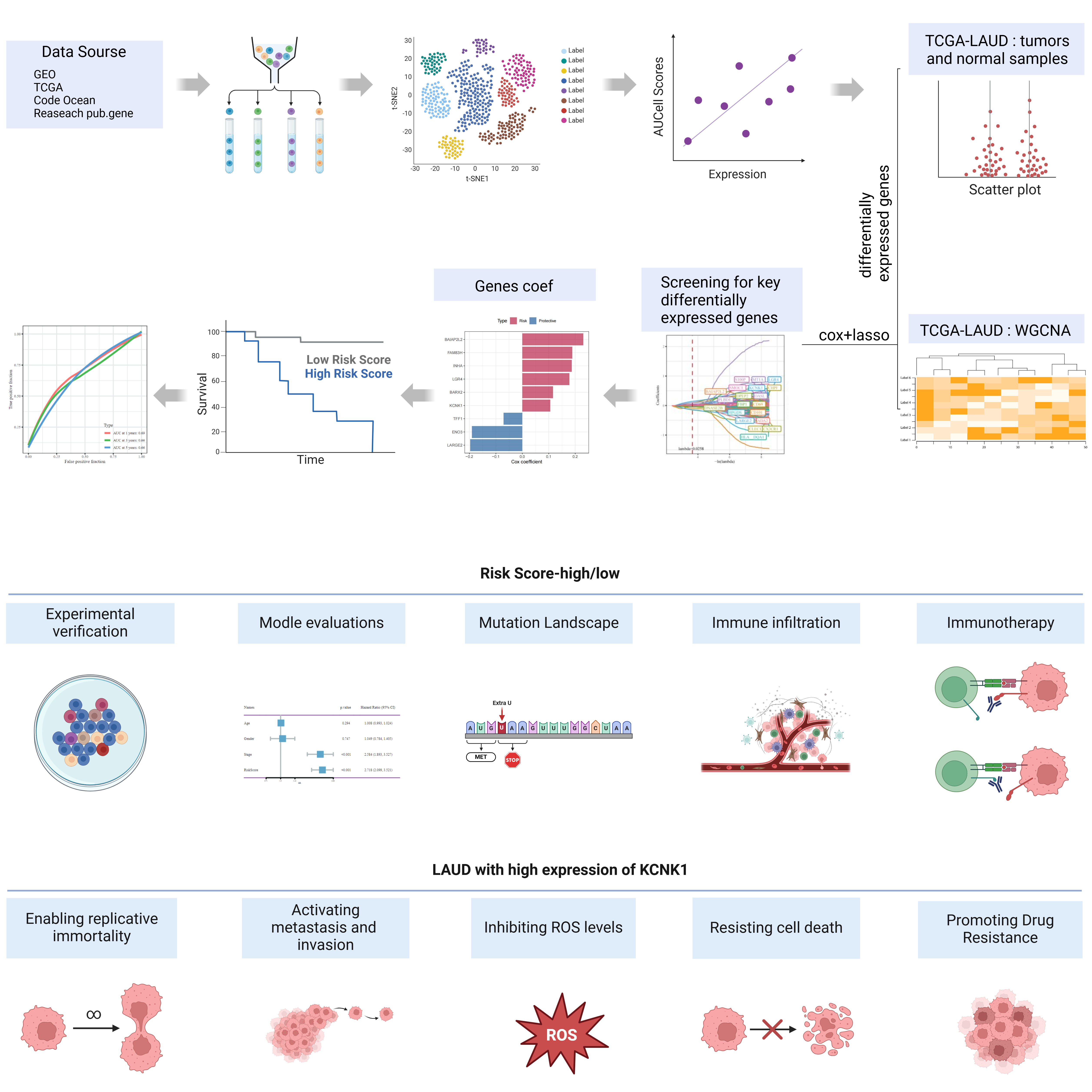

Supplement: Supplementary file 5 [file Image5.tif]

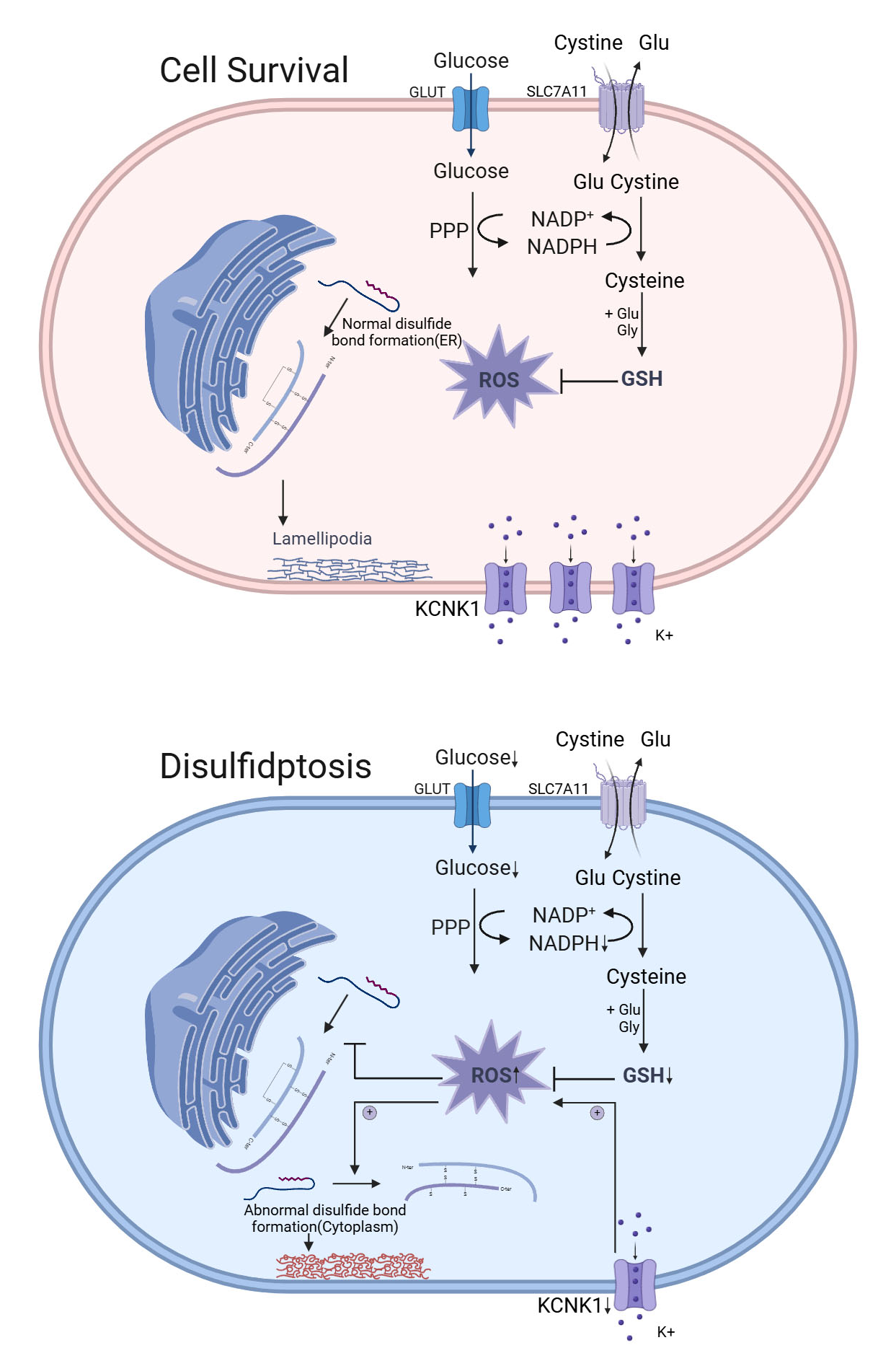

Supplement: Supplementary file 6 [file Image6.tif]
