# Supplementary material for: Toward precision oncology in LUAD: a prognostic model using single-cell sequencing and WGCNA based on a disulfidptosis relative gene signature
Source: Front Immunol. 2025 May 21;16:1581915. doi: 10.3389/fimmu.2025.1581915 (PMC12133857; doi:10.3389/fimmu.2025.1581915)
Supplement: Supplementary file 7 [file Table1.docx]

https://www.jianguoyun.com/p/DbUfaV4Q-fCaDRj96_cFIAA
